# Chemokine–receptor axes in immune–tumor cell communication: spatial niches, immune exclusion, and therapy resistance

**DOI:** 10.3389/fimmu.2026.1879293

**Published:** 2026-10-09

**Authors:** Yimin Han, Qun liu, Fenghua Ao, Jianrui Huang, Hongmin Lin

**Affiliations:** 1 The First Hospital of Putian, Putian, China; 2 Department of Neurosurgery, Heze Municipal Hospital, Heze, Shandong, China; 3 Department of Neurosurgery, The First Affiliated Hospital of Dali University, Dali, Yunnan, China

**Keywords:** chemokine receptors, chemokines, immune exclusion, spatial niches, therapy resistance, tumor microenvironment

## Abstract

Chemokine–receptor axes are traditionally viewed as leukocyte trafficking cues, but increasing evidence indicates that they also function as spatial and functional organizers of immune–tumor communication. In the tumor microenvironment, chemokine gradients determine which immune-cell subsets are recruited, where they are positioned, and whether they acquire antitumor or suppressive states. This review summarizes experimentally validated chemokine–receptor axes that shape spatial niches, immune exclusion, and therapy resistance in cancer. The CXCL12–CXCR4 axis serves as a prototype of stromal immune exclusion, particularly in pancreatic cancer, where CXCR4 blockade can improve immune accessibility but may require myeloid-directed combinations. CXCR2 ligand axes, including CXCL5–CXCR2 and IL-8/CXCR2 signaling, promote neutrophil-rich suppressive niches, CD8^+^ T-cell dysfunction, PD-L1 induction, and resistance to immune checkpoint blockade or chemotherapy. The CCL20–CCR6 axis recruits regulatory T cells and contributes to metastatic immune suppression, whereas CCL5–CCR5 signaling organizes perivascular macrophage niches and supports adaptive therapy resistance. CCL2-associated macrophage circuits further link monocyte recruitment, TAM polarization, and immunotherapy responsiveness. We discuss how single-cell and spatial technologies can validate these circuits and guide biomarker-driven therapies. Rather than targeting isolated ligand–receptor pairs, future strategies should reprogram chemokine-controlled immune ecosystems to overcome immune exclusion and treatment resistance.

## Highlights

- Chemokine–receptor axes spatially organize immune–tumor communication.
- CXCR4, CXCR2, CCR6, CCR5, and CCL2 circuits drive suppressive niches.
- Chemokine-circuit reprogramming may overcome immune exclusion and resistance.

## 1 Introduction

Chemokines are a large family of secreted chemoattractant cytokines that regulate immune-cell migration under homeostatic and inflammatory conditions (1). In classical immunology, chemokines are often described as molecular “navigation signals” that guide leukocytes toward sites of infection, tissue injury, or inflammation. This trafficking function remains fundamental in cancer (2). Tumors exploit chemokine gradients to recruit monocytes, macrophages, neutrophils, regulatory T cells, dendritic cells, and lymphocytes into the tumor microenvironment (3). However, recent evidence indicates that chemokine–receptor axes should not be viewed solely as passive migration cues. They instead function as active organizers of tumor immune architecture, determining not only which immune cells are recruited but also where they are positioned, how long they are retained, and what functional states they acquire after entering tumor tissues (4). This spatial and functional role is particularly important because the tumor microenvironment is highly heterogeneous (5). Cytotoxic T cells may be abundant in the tumor periphery but excluded from tumor-cell nests. Tumor-associated macrophages may accumulate around blood vessels, invasive fronts, or hypoxic regions. Neutrophils and granulocytic myeloid-derived suppressor cells may form local suppressive zones that limit T-cell activation. Regulatory T cells may concentrate in areas enriched in tumor-derived or stromal-derived chemokines. Thus, the presence of immune cells alone does not necessarily indicate effective antitumor immunity. Their spatial distribution and functional orientation are equally important (6). Chemokine–receptor signaling provides one of the major mechanisms through which tumors impose spatial organization on the immune microenvironment (1, 6)—for example, chemokine gradients can direct effector T cells toward or away from tumor-cell nests, retain or position suppressive myeloid populations within stromal compartments, and contribute to the formation of immune-excluded or immune-suppressed regions (1, 7). Spatially resolved studies further demonstrate that chemokine production can organize coordinated intratumoral immune-cell neighborhoods, including T-cell–dendritic-cell microaggregates associated with productive antitumor immunity (4). In this sense, chemokine signaling constitutes a central layer of immune–tumor communication. Tumor cells, stromal cells, endothelial cells, fibroblasts, and diverse immune-cell populations can produce chemokines, while their cognate receptors are expressed across immune, stromal, and malignant-cell compartments, thereby generating reciprocal and context-dependent communication circuits (1). These circuits regulate not only cellular trafficking and positioning but also local survival, proliferation, differentiation, and effector function, ultimately influencing tumor progression, immune evasion, metastatic colonization, and therapeutic response (1, 6). Therefore, chemokine–receptor axes should be considered spatial communication modules rather than simple migration pathways. This conceptual shift is important for understanding why some tumors remain resistant to immune checkpoint blockade despite immune-cell infiltration, why certain tumors show strong myeloid or Treg dominance, and why chemokine-targeted therapies may need to be combined with immune checkpoint inhibitors, chemotherapy, targeted therapy, or myeloid-reprogramming strategies (8).

The tumor immune microenvironment is not uniformly organized. Immune and stromal cells are instead distributed within spatially distinct niches that differ in cellular composition, molecular signaling, metabolic stress, vascular access, extracellular matrix density, and therapeutic vulnerability. These niches include immune-excluded stromal regions, perivascular macrophage niches, neutrophil-rich suppressive zones, Treg-enriched compartments, invasive fronts, hypoxic cores, and metastatic niches (9). Within these regions, chemokine gradients help define the spatial positioning of immune cells and determine whether antitumor immune responses can reach malignant cells effectively. This spatial organization has major biological consequences. A tumor may contain numerous CD8^+^ T cells, but if these cells are trapped in stromal regions or prevented from entering tumor-cell nests, effective tumor killing remains limited (10). Similarly, the accumulation of macrophages or neutrophils near tumor cells, vessels, or stromal barriers can generate local immunosuppressive environments that interfere with T-cell priming, infiltration, or effector function. Tregs may also be recruited into specific compartments, where they suppress cytotoxic lymphocytes and reinforce immune tolerance (11). These observations suggest that immune-cell recruitment must be interpreted together with spatial localization and functional state.

Several chemokine–receptor axes exemplify this spatial logic. The CXCL12–CXCR4 axis is a prototypical mechanism of stromal immune exclusion, particularly in desmoplastic tumors such as pancreatic cancer. CXCL12-enriched stromal regions can restrict effector T-cell access to tumor-cell nests and maintain an immune-excluded phenotype (12). In contrast, CXCR2 ligand axes, including CXCL5–CXCR2 and IL-8/CXCR2 signaling, often promote the accumulation of neutrophils or granulocytic suppressor cells, creating neutrophil-rich niches that impair CD8^+^ T-cell function (13, 14). The CCL20–CCR6 axis can recruit CCR6^+^ regulatory T cells or other suppressive immune subsets, contributing to Treg-enriched immune-suppressive niches in primary and metastatic tumors. Meanwhile, CCL5–CCR5 signaling can organize specialized macrophage-containing niches, including perivascular macrophage compartments that influence immune-cell access and treatment response. Conversely, not all chemokine circuits promote immune suppression. The CXCL16–CXCR6 axis can sustain the retention and survival of effector-like CD8^+^ T cells within supportive intratumoral niches, whereas CXCL9/10/11–CXCR3 signaling promotes trafficking of activated T cells toward tumor regions. Moreover, immune cells frequently co-express multiple chemokine receptors, indicating that their positioning is determined by combinatorial rather than strictly one-to-one ligand–receptor programs (15–17). The emergence of single-cell RNA sequencing, spatial transcriptomics, multiplex immunofluorescence, imaging mass cytometry, and ligand–receptor inference algorithms has made it possible to map these spatial immune circuits with increasing resolution (18). These technologies reveal that chemokine–receptor interactions are not random or globally distributed. They instead often occur in defined tumor regions where specific cellular neighborhoods communicate through short- or long-range signals. This has shifted the field from asking whether a chemokine is expressed in a tumor to asking where it is expressed, which cells produce it, which cells respond to it, and how this communication affects local immune function (19). Understanding chemokine-defined spatial niches is therefore essential for interpreting tumor immunity. It also has therapeutic implications. Targeting a chemokine axis may not simply reduce immune-cell recruitment; it may reorganize immune-cell positioning, break stromal exclusion, disperse suppressive myeloid niches, or allow effector lymphocytes to enter previously inaccessible tumor regions (20).

Resistance to cancer therapy is shaped not only by tumor-intrinsic genetic alterations but also by microenvironmental communication, including chemokine–receptor signaling among tumor, immune, and stromal cells. Chemokine axes can promote resistance to immune checkpoint blockade, chemotherapy, targeted therapy, and combination regimens through several interconnected mechanisms (21). First, chemokine signaling can limit effective T-cell infiltration. In immune-excluded tumors, cytotoxic T cells may remain confined to stromal regions and fail to contact malignant cells, making PD-1/PD-L1 blockade insufficient. The CXCL12–CXCR4 axis in pancreatic cancer and other stromal-rich tumors represents this mechanism (22). Second, chemokines recruit suppressive immune populations. CXCR2-binding ligands attract neutrophils and granulocytic MDSCs, CCL20–CCR6 promotes Treg recruitment, and CCL2-associated pathways sustain monocyte/macrophage accumulation, collectively impairing effector immunity and reducing treatment responsiveness (23). Third, chemokine pathways can directly alter malignant-cell phenotypes, including survival, EMT, immune checkpoint expression, metabolic adaptation, and stress-response programs—for example, CXCL5 may promote PD-L1 expression in lung cancer cells, while CCL5–CCR5 signaling may support tumor-cell survival under therapeutic pressure. Fourth, therapy itself can reshape chemokine networks. Chemotherapy, targeted therapy, radiotherapy, and immunotherapy may induce inflammatory chemokines that either enhance immune recruitment or attract suppressive macrophages, neutrophils, and Tregs, thereby generating adaptive resistance (24). Overall, chemokine–receptor axes connect spatial immune organization, immune-cell composition, tumor-cell adaptation, and treatment-induced remodeling. Targeting these pathways may help convert immune-excluded or immune-suppressed tumors into more therapy-responsive states, but chemokine redundancy and context dependence mean that effective strategies will likely require rational combinations and biomarker-guided patient selection (25).

This review focuses on experimentally validated chemokine–receptor axes that mediate immune–tumor cell communication and shape tumor immune architecture. Rather than providing an exhaustive catalogue of chemokines across all cancer types, we emphasize representative chemokine modules with strong mechanistic, spatial, and therapeutic relevance, including CXCL12–CXCR4, CXCR2 ligand–CXCR2, CCL20–CCR6, CCL5–CCR5, and CCL2-associated macrophage circuits. To broaden tumor-type coverage, we also include selected examples from colorectal, gastric, and ovarian cancers, where chemokine-driven immune suppression, immune exclusion, and therapy resistance have been increasingly characterized. The review is organized around three interconnected themes: how chemokine–receptor axes control immune-cell positioning and spatial niche formation; how they regulate immune exclusion, myeloid/Treg accumulation, CD8^+^ T-cell dysfunction, and tumor-cell immune evasion; and how chemokine signaling contributes to resistance against immune checkpoint blockade, chemotherapy, targeted therapy, and combination strategies. By integrating mechanistic, translational, single-cell, spatial, and therapeutic evidence, we propose that chemokine–receptor axes should be viewed not merely as trafficking cues but as spatial and functional communication modules that determine whether the tumor microenvironment supports immune surveillance or therapy-resistant immune escape.

## 2 Conceptual framework: how chemokine–receptor axes control immune–tumor communication

Chemokine–receptor axes provide a useful conceptual framework for understanding how immune and tumor cells communicate within the tumor microenvironment. Traditionally, chemokines have been interpreted mainly as chemoattractant signals that guide immune cells into tumor tissues. However, this view is incomplete. In cancer, chemokine signaling operates at multiple levels: it determines which immune-cell subsets are recruited, whether they persist within the tissue, where they are positioned within tumor niches, and how their functional states are shaped after arrival (1). At the same time, chemokine receptors expressed by malignant cells can directly activate signaling programs that regulate tumor-cell survival, proliferation, invasion, metastatic behavior, immune evasion, and therapeutic adaptation (26, 27). Therefore, chemokine–receptor axes should be understood as multilayered communication systems rather than linear recruitment pathways. A single chemokine axis may simultaneously influence immune-cell trafficking, retention, spatial localization, suppressive niche formation, and tumor-cell adaptation—for example, CXCL5–CXCR2 signaling promotes the accumulation of tumor-associated neutrophils in lung cancer, and these neutrophil-rich environments impair the differentiation and effector function of antitumor CD8^+^ T cells and reduce responsiveness to immune checkpoint blockade (28). Similarly, CXCL12–CXCR4 signaling can regulate immune-cell positioning in stromal-rich tumors; in pancreatic cancer, fibroblast-derived CXCL12 contributes to T-cell exclusion from malignant-cell regions, whereas CXCR4 inhibition promotes T-cell accumulation within tumor-cell areas and enhances checkpoint blockade in preclinical models (29). CCL20–CCR6 signaling can selectively recruit CCR6^+^ regulatory T cells into tumors, thereby reinforcing local immunosuppressive niches (30). In contrast, CCL5–CCR5 signaling can exert multiple context-dependent effects. CCR5-dependent LYVE-1^+^ macrophages can form specialized perivascular niches that influence chemotherapy responses (31), whereas CCL5–CCR5 signaling can also directly activate adaptive survival programs in malignant cells under therapeutic pressure, including a recently described CCL5–CCR5–CYP1A1 pathway associated with resistance to lenvatinib-based therapy in hepatocellular carcinoma (32). Based on these observations, this review proposes a four-layer model of chemokine–receptor regulation in immune–tumor communication: a trafficking layer, which determines which cells enter the tumor; a retention layer, which determines whether recruited cells persist within the tissue; a spatial-positioning layer, which determines where these cells localize and which cellular partners they encounter; and a functional layer, which determines how chemokine signaling reshapes the phenotype and activity of immune, stromal, and malignant cells.

### 2.1 A four-layer framework: trafficking, retention, spatial positioning, and function

#### 2.1.1 Trafficking layer: determining which immune cells enter the tumor

The first and most established layer of chemokine–receptor regulation is immune-cell trafficking. Chemokines produced by tumor cells, stromal cells, endothelial cells, cancer-associated fibroblasts, and resident immune cells create gradients that attract circulating or tissue-resident immune populations into tumor tissues. This process determines the initial cellular composition of the tumor microenvironment. Importantly, immune-cell recruitment is not inherently beneficial or harmful. Its biological consequence depends on which immune-cell subsets are recruited. Recruitment of cytotoxic CD8^+^ T cells, natural killer cells, and type 1 dendritic cells may enhance antitumor immunity. In contrast, recruitment of regulatory T cells, suppressive macrophages, neutrophils, or myeloid-derived suppressor cells may reinforce immune escape (33). Thus, chemokine–receptor axes act as a selective filter that shapes the balance between immune activation and immune suppression.

Several axes illustrate this trafficking layer. CXCL5/CXCL8–CXCR2 signaling preferentially recruits neutrophils and granulocytic myeloid-derived suppressor cells. These cells frequently generate a suppressive myeloid compartment that limits T-cell function and contributes to immunotherapy resistance. In lung cancer, CXCL5-mediated neutrophil accumulation has been linked to impaired anticancer CD8^+^ T-cell differentiation and reduced checkpoint inhibitor efficacy (34). In hepatocellular carcinoma, IL-8/CXCR2 signaling similarly sustains myeloid-driven immune resistance. The CCL20–CCR6 axis is another trafficking module, often associated with recruitment of CCR6^+^ regulatory T cells or other suppressive lymphocyte subsets. Tumor-derived or macrophage-derived CCL20 can attract CCR6-expressing Tregs, thereby promoting local immune suppression. This mechanism is especially relevant in tumors where Treg accumulation limits cytotoxic immune responses.

The CCL2-associated axis primarily controls monocyte and macrophage trafficking. CCL2 can recruit CCR2^+^ monocytes into tumor tissues, where they may differentiate into tumor-associated macrophages. These macrophages can then support angiogenesis, tissue remodeling, immune suppression, and therapeutic resistance. In macrophage-rich tumors, CCL2-related signaling is therefore a major determinant of myeloid composition (35). The CXCL12–CXCR4 axis also contributes to trafficking, but its effect is more complex. Rather than simply attracting effector lymphocytes into tumor nests, CXCL12–CXCR4 signaling can retain immune cells in stromal regions or contribute to immune exclusion. In this way, the same chemokine axis can influence both recruitment and spatial restriction (36). These examples together show that the trafficking layer defines the immune-cell input into tumors. It answers the first question in chemokine-mediated immune–tumor communication: which immune cells are recruited into the tumor microenvironment?

#### 2.1.2 Retention layer: CXCL16–CXCR6 and intratumoral T-cell persistence

The second layer is immune-cell retention, which determines whether recruited lymphocytes persist within functionally productive intratumoral niches after initial entry. This distinction is particularly important for cytotoxic T cells because effective antitumor immunity requires not only trafficking into tumors but also sustained local residence, survival, and interaction with supportive cellular partners. Tissue-resident memory T (T_RM) cells represent a specialized memory population adapted for long-term persistence in peripheral tissues, where they provide rapid local immune surveillance. Studies and reviews from Farber and colleagues have established tissue residency as a distinct immunological program and highlighted chemokine receptors, including CXCR6, as important components of tissue-specific T-cell localization and maintenance (37, 38). In the human lung, CXCR6 has also been associated with tissue-resident T-cell phenotypes, supporting its broader role in the compartmentalization of local memory responses (39). This relationship is also evident in cancer. In human non-small cell lung cancer, tumor-infiltrating CD8^+^ T cells with strong T_RM-associated transcriptional features showed increased CXCR6 expression, and greater enrichment of T_RM-like cytotoxic lymphocytes was associated with more robust antitumor immunity and favorable clinical outcome (40). Functional evidence from ovarian cancer further demonstrated that CXCR6 promotes the retention of tumor-specific memory CD8^+^ T cells within tumor tissues; CXCR6 deficiency reduced intratumoral persistence, weakened resident memory responses, and impaired tumor control (41). These observations indicate that CXCR6 is not merely a phenotypic marker of tissue-associated T cells but can actively contribute to their localization and persistence within the tumor microenvironment.

Mechanistically, the CXCL16–CXCR6 axis provides a particularly illustrative example of how chemokine signaling couples T-cell retention to spatially defined supportive niches. Di Pilato et al. demonstrated that effector-like cytotoxic T lymphocytes upregulate CXCR6 and are positioned within discrete perivascular stromal niches enriched in CCR7^+^ dendritic cells expressing CXCL16. These dendritic cells provide IL-15 through trans-presentation, thereby supporting the local survival and expansion of CXCR6^+^ effector-like CD8^+^ T cells and sustaining antitumor activity before terminal dysfunction develops (15). Consistently, CXCR6 has been shown to be required for the antitumor efficacy of intratumoral CD8^+^ T cells and for optimal responsiveness to PD-1 blockade in experimental tumor models (42).

Thus, CXCL16–CXCR6 signaling should not be interpreted solely as a conventional recruitment pathway. It instead represents a spatial retention and survival module that links tissue-residency programs with chemokine-guided positioning and access to trophic signals within specialized immune-supportive niches. Importantly, CXCR6 expression alone should not be considered sufficient to define T_RM identity, which is determined by integrated phenotypic, transcriptional, functional, and anatomical features (37). Nevertheless, accumulating evidence supports CXCR6 as an important regulator of T_RM-associated localization, persistence, and antitumor function within selected tumor contexts.

#### 2.1.3 Spatial layer: determining where immune cells are positioned

The third layer is spatial organization. After immune cells enter the tumor microenvironment, their position relative to malignant cells, stromal barriers, vasculature, extracellular matrix, and other immune populations strongly influences their function. The same immune-cell subset may have different biological effects depending on its localization (43)—for example, CD8^+^ T cells located at the tumor margin or trapped in stromal compartments may be less effective than CD8^+^ T cells that directly infiltrate tumor-cell nests. Similarly, macrophages located around vessels, invasive fronts, or hypoxic regions may form specialized niches with distinct functions (44).

Chemokine gradients are central to this spatial organization. They help determine whether immune cells penetrate tumor-cell areas, remain excluded in the stroma, cluster around blood vessels, or accumulate in metastatic sites. This spatial layer is particularly important for understanding immune-excluded tumors, in which immune cells are present but fail to contact malignant cells effectively. The CXCL12–CXCR4 axis is a prototypical spatial exclusion mechanism. In stromal-rich tumors such as pancreatic cancer, CXCL12-rich compartments can prevent effector T cells from entering tumor-cell nests. T cells may instead remain trapped in fibroblast-rich or stromal regions, producing an immune-excluded phenotype (45). Therapeutic CXCR4 blockade can partially reverse this pattern by increasing T-cell access to tumor regions, although compensatory myeloid accumulation may still limit durable responses.

CCR5-dependent signaling provides another example of spatial regulation. In breast cancer, LYVE-1^+^ tumor-associated macrophages form coordinated multicellular structures preferentially located near tumor vasculature, and the formation of these perivascular macrophage niches is dependent on CCR5 signaling (31). Disruption of LYVE-1^+^ macrophage development or their CCR5-dependent spatial organization increases CD8^+^ T-cell recruitment and enhances chemotherapy responsiveness, demonstrating that chemokine signaling can regulate not only macrophage abundance but also their anatomical organization and functional interaction with antitumor lymphocytes (31).

The CCL20–CCR6 axis may also contribute to spatially restricted immunosuppressive microenvironments, particularly in metastatic or Treg-enriched tumor compartments. In human prostate cancer bone metastases, single-cell profiling identified myeloid cells as a major source of CCL20 and T cells as prominent CCR6-expressing responders. Functional disruption of the CCL20–CCR6 axis restored T-cell reactivity and prolonged survival in preclinical bone-metastasis models, supporting its role in maintaining an immunosuppressive metastatic niche (46). In hepatocellular carcinoma, CCR6^+^ regulatory T cells preferentially accumulate within tumor tissues, where their distribution correlates with intratumoral CCL20 expression, providing evidence that CCL20–CCR6 signaling contributes to the selective localization of suppressive Tregs within the tumor microenvironment (30). Recent evidence in pancreatic cancer further demonstrates that tumor-derived CCL20 recruits CCR6^+^ Tregs, reduces productive CD8^+^ T-cell infiltration, and promotes an immunosuppressive microenvironment, whereas genetic or pharmacological disruption of CCR6 enhances antitumor immunity and responsiveness to PD-1 blockade (47). These studies together illustrate that chemokines function not only as recruitment signals but also as spatial organizers that influence where responding immune cells accumulate, which cellular partners they encounter, and whether immune effector cells can gain productive access to malignant-cell regions (1). This spatial layer therefore addresses a central question in chemokine-mediated immune–tumor communication: where are recruited or retained immune cells positioned within tumor tissue, and how does that localization influence their function?

#### 2.1.4 Direct cellular effects of chemokine–receptor signaling

The fourth layer is the direct cellular-response layer. In this layer, chemokine–receptor axes are interpreted not only as indirect regulators of the tumor microenvironment through immune-cell recruitment, but also as direct signaling modules that alter the behavior of receptor-expressing cells. When chemokines bind their cognate receptors, they activate intracellular signaling programs that can regulate migration, adhesion, survival, proliferation, polarization, exhaustion, cytokine production, checkpoint expression, epithelial–mesenchymal transition, metabolic adaptation, and stress responses (23). These direct effects may occur in malignant cells, T cells, neutrophils, macrophages, dendritic cells, endothelial cells, or fibroblasts, depending on the cellular distribution of the corresponding receptor—for example, CXCR2 signaling regulates both the recruitment and functional state of tumor-associated neutrophils and other suppressive myeloid populations. Genetic or pharmacological inhibition of CXCR2 can reduce myeloid-mediated tumor progression and enhance T-cell infiltration, whereas recent functional studies further indicate that CXCR1/2 blockade can suppress neutrophil immunosuppressive programs, including ROS and arginase-1 production (48, 49). CXCL5 can additionally exert tumor-cell-intrinsic effects: in lung cancer cells, CXCL5 was shown to increase PD-L1 expression through PXN/AKT signaling while simultaneously promoting neutrophil chemotaxis and CD8^+^ T-cell dysfunction (50).

CCL20–CCR6 signaling also acts directly on CCR6^+^ Tregs. Experimental and clinical studies have demonstrated that CCL20 promotes selective recruitment of CCR6^+^ Tregs into tumors, while more recent work indicates that CCR6–CCL20 signaling can additionally reinforce the glycolytic and immunosuppressive state of tumor-infiltrating Tregs (8, 30). CCL5–CCR5 signaling may likewise directly affect CCR5^+^ malignant cells under therapeutic pressure. In hepatocellular carcinoma, treatment-induced CCL5 derived in part from CD8^+^ T cells was reported to activate a CCR5–CYP1A1 program in tumor cells, thereby promoting resistance to lenvatinib and combined anti-PD-1/lenvatinib therapy (32). Because this specific adaptive-resistance mechanism is currently supported primarily by one mechanistic study, it should be regarded as preliminary and requires validation in independent models and clinical cohorts. Therefore, distinguishing direct receptor-mediated cellular effects from indirect niche-level consequences is essential for interpreting how chemokine axes shape immune exclusion, cellular adaptation, and therapy resistance.

### 2.2 Effector-supportive chemokine programs and chemokine-receptor co-expression

CXCL9/10/11–CXCR3 signaling represents another major chemokine program supporting productive antitumor immunity. CXCR3 is expressed by activated and effector CD8^+^ and CD4^+^ T cells and responds to the interferon-inducible ligands CXCL9, CXCL10, and CXCL11. In contrast to chemokine circuits that predominantly recruit suppressive myeloid cells or Tregs, CXCR3 signaling frequently facilitates effector T-cell trafficking toward tumor regions and contributes to the formation of T-cell-inflamed microenvironments. Macrophage-derived CXCL9 and CXCL10 have been shown to be required for CXCR3-dependent CD8^+^ T-cell recruitment and therapeutic efficacy following immune checkpoint blockade (16). Similarly, increased CXCL9/10 production by macrophages and fibroblasts can enhance CD8^+^ T-cell recruitment into pancreatic tumors (51). Conversely, suppression of CXCL9, CXCL10, and CXCL11 can contribute to impaired T-cell infiltration, as demonstrated in colorectal cancer models in which restoration of this chemokine program increased CD8^+^ T-cell infiltration in a CXCR3-dependent manner (52). These findings illustrate that chemokine networks can generate not only suppressive or immune-excluded niches but also spatial programs that promote productive lymphocyte access and antitumor immunity.

### 2.3 A working model for this review

Based on the four-layer framework described above, this review proposes that chemokine–receptor networks function as combinatorial spatial communication modules integrating immune-cell recruitment, retention, spatial positioning, and functional reprogramming within the tumor microenvironment (1). In this model, chemokine–receptor signaling does not merely determine whether immune cells enter tumors. It also influences whether recruited cells persist within the tissue, where they are positioned relative to malignant, stromal, vascular, and immune compartments, which cellular partners they encounter, and which functional states they subsequently acquire (1). Thus, a chemokine signal may recruit an immune population, retain or position it within a specific niche, and reinforce a functional state that promotes either immune surveillance or immune escape.

Several representative axes illustrate this multilayered organization. CXCL12–CXCR4 signaling can establish an immune-excluded stromal architecture by restricting effective T-cell access to tumor-cell regions; in pancreatic cancer models, inhibition of CXCR4 promotes rapid T-cell accumulation among malignant cells and enhances the activity of PD-L1 blockade (29). CXCR2 ligand axes can recruit and regulate neutrophils and granulocytic suppressor populations, generating myeloid-dominated environments that impair CD8^+^ T-cell differentiation and function and limit responsiveness to immune checkpoint blockade (28, 53). CCL20–CCR6 signaling can promote the accumulation of CCR6^+^ regulatory T cells and contribute to immunosuppressive compartments in both primary tumors and metastatic sites, as demonstrated in hepatocellular carcinoma and prostate cancer bone metastases (30, 46). CCL5–CCR5 signaling can organize specialized LYVE-1^+^ perivascular macrophage niches that influence CD8^+^ T-cell access and chemotherapy response (31), while tumor-cell-intrinsic CCL5–CCR5 signaling may also activate adaptive survival programs under therapeutic pressure, including the recently described CCR5–CYP1A1 pathway in hepatocellular carcinoma (32). CCL2-associated signaling similarly regulates monocyte/macrophage recruitment and polarization; in hepatocellular carcinoma, SLAMF7-regulated CCL2 signaling promotes immunosuppressive macrophage states and influences responsiveness to PD-1 blockade (54).

Importantly, chemokine-controlled spatial organization is not exclusively immunosuppressive. CXCL16–CXCR6 signaling can position effector-like CD8^+^ T cells within CXCL16-enriched perivascular dendritic-cell niches that provide IL-15-dependent survival signals and sustain local antitumor activity (15). The CXCL9/10/11–CXCR3 axis likewise promotes recruitment of activated effector T cells into tumor tissues and is required for effective CD8^+^ T-cell infiltration and therapeutic responses to immune checkpoint blockade in several experimental and clinical settings (16). These effector-supportive pathways emphasize that the biological consequence of chemokine signaling depends on the identity and state of the receptor-expressing cell, the cellular source of the ligand, and the spatial context in which the interaction occurs (1). This framework also helps explain why targeting a single chemokine axis may produce incomplete therapeutic benefit. Blocking one receptor may improve effector-cell accessibility while simultaneously allowing compensatory suppressive populations or alternative chemokine circuits to become dominant—for example, clinical CXCR4 antagonism in metastatic pancreatic cancer increased intratumoral T-cell recruitment but was also associated with enrichment of immunosuppressive macrophages, illustrating that relief of one spatial barrier does not necessarily produce durable antitumor immunity (55). Similarly, disrupting immune exclusion may remain insufficient when tumor cells retain intrinsic survival programs or engage alternative immunosuppressive pathways. Chemokine-targeted therapies should therefore be developed with attention to ligand source, receptor-expressing cell population, spatial niche, treatment context, and compensatory immune circuits (1, 55).

Accordingly, the central premise of this review is that chemokine–receptor networks regulate four interconnected dimensions of immune–tumor communication: which immune cells are recruited, whether they are retained, where they are positioned, and how their functional states influence tumor immunity and therapeutic response. This framework provides the basis for discussing individual chemokine modules in the following sections and for evaluating their therapeutic potential in immune exclusion and treatment resistance. Representative experimentally validated chemokine–receptor axes involved in immune–tumor communication are summarized in **Table 1**. These studies illustrate how distinct chemokine modules regulate immune-cell recruitment and retention, spatial positioning, suppressive or effector-supportive niche formation, tumor-cell adaptation, and therapy response across different cancer contexts. As illustrated in **Figure 1**, chemokine–receptor networks should therefore be viewed as multilayered spatial communication systems rather than simple immune-cell trafficking pathways.

**Table 1:** Experimentally validated chemokine–receptor axes in immune–tumor communication.

Chemokine–receptor axis	Cancer type / model	Main ligand source	Receptor-expressing cells	Major immune / spatial outcome	Therapy relevance	PMID
CXCL12–CXCR4	Pancreatic cancer mouse model	FAP^+^ carcinoma-associated fibroblasts	CXCR4^+^ T cells	CXCL12-mediated stromal T-cell exclusion from cancer-cell regions	CXCR4 inhibition with AMD3100 synergizes with anti-PD-L1 therapy	24277834
CXCL12–CXCR4	Metastatic pancreatic cancer	Stromal / tumor-associated niche components	CXCR4^+^ immune cells and tumor-associated cells	Stromal immune exclusion; restricted T-cell access to tumor regions	CXCR4 antagonist BL-8040 combined with pembrolizumab and chemotherapy remodels the immune microenvironment	32451495
CXCL12–CXCR4	Metastatic pancreatic adenocarcinoma	Stromal compartments	T cells and macrophage-lineage cells	T-cell recruitment accompanied by compensatory immunosuppressive macrophage accumulation	CXCR4 antagonism plus PD-1 inhibition may require additional myeloid-targeted strategies	40815609
CXCR2 ligand–CXCR2	NASH-associated HCC	Inflammation-associated tumor / stromal compartments	CXCR2^+^ neutrophils and myeloid cells	Neutrophil-rich inflammatory niche; immune checkpoint resistance	CXCR2 inhibition enables immunotherapy responsiveness in NASH-HCC	35477863
IL-8/CXCR2	HCC	Myeloid / tumor-associated inflammatory compartments	CXCR2^+^ suppressive myeloid cells	Myeloid immune suppression; impaired antitumor immunity	Targeting IL-8/CXCR2 signaling overcomes immunotherapy resistance	39916327
CXCL5–CXCR2	Lung cancer	Tumor cells	CXCR2^+^ mature neutrophils	Neutrophil accumulation; impaired CD8^+^ T-cell differentiation	Limits checkpoint inhibitor efficacy	35402081
CXCL5–CXCR2	Lung cancer	Tumor cells	CXCR2^+^ neutrophils; CXCL5-responsive tumor cells	Neutrophil chemotaxis; PD-L1 upregulation via PXN/AKT signaling	Suppresses CD8^+^ T-cell immunity and promotes immune escape	39034411
CXCL5–CXCR2	Obesity-associated pancreatic cancer	Tumor cells	CXCR2^+^ myeloid cells / immune populations	Reduced T-cell infiltration in obesity-shaped immune niches (preclinical model)	In a preclinical obese PDAC model, CXCL5 depletion improved T-cell infiltration and anti-PD-1 response; human validation is needed	40121029
CXCR2-associated axis	Lung cancer	Tumor and stromal compartments	CXCR2^+^ tumor cells and neutrophils	Tumor progression, neutrophil infiltration, EMT-related programs	CXCR2 targeting enhances cisplatin efficacy	33814009
CCL20–CCR6	Prostate cancer bone metastasis	Myeloid cells	CCR6^+^ T cells	Immunosuppressive metastatic bone niche; reduced T-cell reactivity	Disrupting CCL20–CCR6 restores T-cell reactivity and prolongs survival in models	34719426
CCL20–CCR6	Pancreatic cancer	Tumor cells	CCR6^+^ Tregs	Treg-rich immunosuppressive niche	Targeting tumor-derived CCL20 may reduce Treg recruitment	41643988
CCL20–CCR6	HCC	Galectin-1-induced TAMs	CCR6^+^ Tregs	TAM-driven Treg recruitment; suppressed antitumor immunity	Targeting Galectin-1 reduces CCL20 secretion and Treg recruitment	39853961
CCL5–CCR5	BAP1-mutant clear cell renal cell carcinoma	Tumor / immune microenvironment	CCR5^+^ immune cells	Immunosuppressive tumor microenvironment shaped by genetic context	CCR5 blockade enhances antitumor immunity	32371459
CCL5–CCR5	Breast cancer	Perivascular niche-associated cells	CCR5-dependent LYVE-1^+^ macrophages	Perivascular macrophage niche; altered CD8^+^ T-cell recruitment	Influences chemotherapy response	37442140
CCL5–CCR5–CYP1A1	Liver cancer	Tumor / microenvironmental CCL5 source	CCR5^+^ liver cancer cells	Tumor-cell survival under therapeutic pressure	Promotes resistance to combined targeted and immune-based therapies	39183447
SLAMF7-regulated CCL2 signaling	HCC	Tumor / immune regulatory compartments	Monocytes / macrophages	Immunosuppressive TAM state; macrophage-dominated immune suppression	Targeting SLAMF7-regulated CCL2 signaling repolarizes macrophages and sensitizes HCC to immunotherapy	38484085
CCL20–CCR6	5-FU-resistant colorectal cancer	5-FU-resistant CRC cells	CCR6^+^ Tregs	Treg recruitment; immunosuppressive chemoresistance niche	Promotes chemoresistance via FOXO1/CEBPB/NF-κB signaling	31395078
CCL20–CCR6	Colorectal cancer	RANKL/RANK-activated tumor cells	CCR6^+^ Tregs	Treg recruitment; stemness and metastatic progression	Links RANK signaling to CCL20–CCR6-mediated immune suppression and metastasis	38902257
CCL5–CCR5	Colorectal cancer liver metastasis	Invasive-margin / tumor-associated immune cells	CCR5^+^ immune and myeloid cells	Tumor-promoting inflammatory niche; macrophage repolarization	Maraviroc-mediated CCR5 blockade showed early clinical antitumor immune remodeling	27070705
CXCL8–CXCR1/2	Gastric cancer	TAMs	CXCR1/2-expressing macrophage / tumor-associated cells	PD-L1^+^ macrophage-associated immune evasion	CXCR1/2 inhibition with reparixin reduces PD-L1^+^ macrophages and promotes antitumor immunity	30661053
CCL20–CCR6	Gastric adenocarcinoma	PPARδ-activated gastric epithelial / tumor cells	CCR6^+^ immune cells	Gastric TME remodeling; immunosuppressive immune-cell infiltration	PPARδ inhibition suppresses CCL20/CCR6-mediated immune remodeling	37572185
CXCL12–CXCR4	Gastric cancer	Tumor / stromal CXCL12 source	CXCR4^+^ TAMs and tumor cells	PD-L1 upregulation; M2-like TAM polarization	Confers anti-PD-1 resistance via CXCL12/CXCR4/PI3K/AKT/HIF-1α signaling	42310180
CCL2-associated axis	Ovarian cancer	Cancer-associated mesenchymal stromal cells	CCR2hi monocytes / macrophages	CD8^+^ T-cell exclusion; suppressive macrophage polarization	Hedgehog inhibition reverses immune exclusion and improves anti-PD-L1 response in models	34767446
MYBL2–CCL2	Ovarian cancer	MYBL2-high tumor cells	TAMs / macrophages	TAM recruitment and M2-like polarization	Promotes anti-PD-1 resistance; potential target to enhance immunotherapy	37865750
CCL2-associated axis	Ovarian cancer	CCL2-overexpressing ovarian cancer cells	Macrophages and tumor cells	Autocrine survival signaling and macrophage recruitment	Associated with paclitaxel resistance	36076499

**Figure 1:**
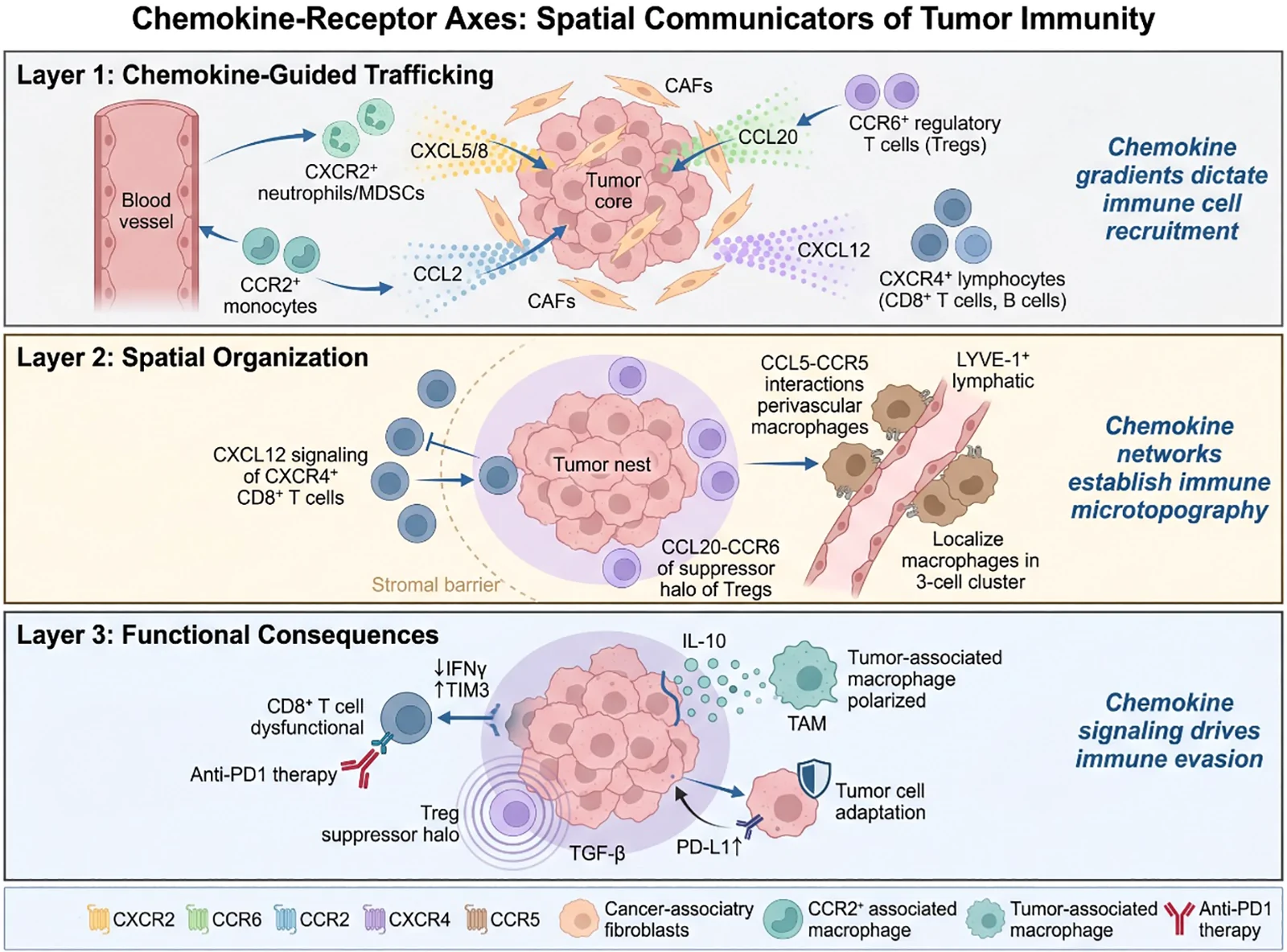
Conceptual framework of chemokine–receptor axes in immune–tumor communication. Chemokine gradients regulate three interconnected layers: immune-cell trafficking, spatial positioning within tumor niches, and functional reprogramming of immune and tumor cells. Representative axes, including CXCL12–CXCR4, CXCR2 ligand–CXCR2, CCL20–CCR6, CCL5–CCR5, and CCL2–CCR2, coordinate immune exclusion, suppressive niche formation, tumor adaptation, and therapy resistance.

## 3 CXCL12–CXCR4 axis: the prototype of stromal immune exclusion

### 3.1 Biological role of CXCL12–CXCR4 in tumor immune exclusion

The CXCL12–CXCR4 axis is one of the best-characterized chemokine–receptor systems involved in tumor progression, metastatic dissemination, and immune escape. CXCL12, also known as stromal cell-derived factor 1, is produced by a variety of non-malignant cells within the tumor microenvironment, including stromal cells, cancer-associated fibroblasts, endothelial cells, perivascular cells, and other tumor-associated niche components (56). Its receptor, CXCR4, is expressed by multiple immune-cell subsets as well as by many types of malignant cells. This broad cellular distribution allows CXCL12–CXCR4 signaling to regulate both immune-cell positioning and tumor-cell behavior.

In the context of tumor immunity, the CXCL12–CXCR4 axis is particularly important because it can create a stromal immune-exclusion barrier. In stromal-rich tumors such as pancreatic ductal adenocarcinoma, effector T cells may be present in the tumor microenvironment but remain spatially separated from malignant epithelial cells. Instead of penetrating tumor-cell nests, T cells are often retained in fibroblast-rich or stromal compartments. This pattern defines an immune-excluded phenotype, in which antitumor lymphocytes are present but cannot effectively contact and eliminate tumor cells.

Mechanistically, CXCL12 gradients can contribute to this exclusion by guiding or retaining CXCR4-expressing immune cells within stromal regions. Dense extracellular matrix, activated fibroblasts, abnormal vasculature, and myeloid-rich inflammation further reinforce this barrier (57). Therefore, CXCL12–CXCR4 signaling does not simply regulate immune-cell recruitment; it controls the spatial accessibility of immune cells to tumor-cell nests. This distinction is important because immune checkpoint inhibitors require pre-existing or inducible T-cell access to malignant cells. If T cells are trapped outside tumor nests, blockade of PD-1 or PD-L1 alone may be insufficient. The CXCL12–CXCR4 axis may also influence tumor-cell survival, invasion, and metastatic tropism. Many tumor cells express CXCR4 and can respond to CXCL12-rich niches in distant organs, contributing to metastatic colonization. However, in the context of this review, its most relevant function is its ability to organize stromal immune exclusion and limit productive immune–tumor cell contact. For this reason, CXCL12–CXCR4 can be considered a prototypical chemokine axis linking stromal architecture, immune-cell positioning, and resistance to immune checkpoint blockade. A foundational preclinical study by Feig and colleagues provided the mechanistic basis for this concept in pancreatic ductal adenocarcinoma. Using a genetically engineered mouse model of pancreatic cancer, they showed that FAP-expressing carcinoma-associated fibroblasts produced CXCL12, which contributed to the exclusion of T cells from cancer-cell regions. Pharmacological inhibition of CXCR4 with AMD3100 induced rapid T-cell accumulation among malignant cells and synergized with anti-PD-L1 therapy to reduce tumor burden. This study established CXCL12–CXCR4 signaling as a stromal immune-exclusion mechanism and provided an important mechanistic rationale for subsequent clinical testing of CXCR4 blockade combined with checkpoint inhibition (29).

### 3.2 Clinical evidence from pancreatic cancer

Pancreatic ductal adenocarcinoma provides one of the clearest examples of CXCL12–CXCR4-mediated stromal immune exclusion, with both foundational preclinical support and subsequent clinical translation. PDAC is characterized by dense desmoplastic stroma, poor effector T-cell infiltration into tumor-cell nests, abundant suppressive myeloid populations, and limited responsiveness to immune checkpoint blockade (29, 55, 58). Building on preclinical evidence that CXCR4 blockade can overcome CXCL12-dependent T-cell exclusion and synergize with checkpoint inhibition, the COMBAT trial, reported by Bockorny and colleagues in *Nature Medicine* in 2020, evaluated the CXCR4 antagonist BL-8040 in combination with pembrolizumab, with or without chemotherapy, in patients with metastatic pancreatic cancer. This study evaluated the CXCR4 antagonist BL-8040 in combination with pembrolizumab, with or without chemotherapy, in patients with metastatic pancreatic cancer. This clinical strategy directly extended the preclinical rationale that CXCR4 inhibition can relieve CXCL12-mediated immune exclusion and enhance checkpoint blockade efficacy. The trial showed that BL-8040 combined with pembrolizumab and chemotherapy could remodel the immune microenvironment of metastatic pancreatic cancer and provided clinical evidence supporting CXCR4 inhibition as part of an immune-sensitizing strategy (58). In cohort 2, patients received BL-8040 and pembrolizumab with chemotherapy, and the study reported clinically meaningful antitumor activity, supporting the translational relevance of CXCR4 blockade in PDAC.

This finding is important for several reasons. First, it supports the concept that immune resistance in PDAC is due not only to absence of tumor antigens or checkpoint expression but also to spatial exclusion of immune cells. Second, it suggests that chemokine-targeted therapy may convert an immune-excluded tumor into a more immune-accessible tumor. Third, it provides a rationale for combining CXCR4 antagonists with PD-1 blockade and cytotoxic chemotherapy. Chemotherapy may increase antigen release, tumor-cell stress, and inflammatory signaling, while CXCR4 blockade may improve immune-cell access and PD-1 blockade may reinvigorate exhausted T cells. In this sense, the COMBAT trial illustrates a combination strategy designed to overcome several layers of immune resistance simultaneously.

A subsequent phase 2 study by Bever and colleagues further refined this concept by examining CXCR4 antagonism combined with PD-1 inhibition in metastatic pancreatic adenocarcinoma (55). The study showed that CXCR4 antagonism could recruit T cells into the tumor microenvironment, supporting the idea that CXCR4 blockade can alter immune-cell trafficking and accessibility. However, the immune remodeling was more complex than a simple conversion from “cold” to “hot” tumor. The treatment was also associated with recruitment or expansion of immunosuppressive macrophages, suggesting that disruption of CXCR4-mediated exclusion may trigger compensatory myeloid programs. This observation has important implications for therapeutic design. It indicates that improving T-cell recruitment alone may not be sufficient if suppressive macrophages, neutrophils, or other myeloid populations simultaneously accumulate. In other words, breaking a stromal chemokine barrier may increase immune-cell access but may not guarantee effective antitumor immunity. The quality, localization, and suppressive potential of recruited immune cells must also be considered. Therefore, CXCR4 blockade may require rational combination with macrophage-targeting or myeloid-reprogramming strategies, such as CSF1R inhibition, CCR2 blockade, CD40 agonism, TLR agonists, or other approaches that reduce suppressive myeloid activity. These pancreatic cancer studies together demonstrate both the promise and limitation of targeting CXCL12–CXCR4 signaling. On the one hand, CXCR4 antagonism can remodel immune architecture and promote T-cell recruitment. On the other hand, compensatory immunosuppressive macrophage accumulation may blunt therapeutic efficacy. This dual outcome reinforces the broader theme of this review: chemokine–receptor axes must be interpreted as part of dynamic spatial and cellular networks rather than as isolated ligand–receptor pairs.

Despite these encouraging translational findings, an important clinical gap remains. CXCR4 antagonists have achieved regulatory approval in settings distinct from solid-tumor immune modulation—for example, plerixafor and motixafortide are used for hematopoietic stem-cell mobilization, whereas the oral CXCR4 antagonist mavorixafor has been approved for WHIM syndrome, a CXCR4-driven primary immunodeficiency (59–61). However, no CXCR4 antagonist has yet been approved as an immune-modulatory antitumor therapy for solid malignancies. Therefore, the COMBAT trial should be interpreted as proof-of-concept translational evidence rather than definitive clinical validation of CXCR4 blockade as an established immunotherapy-sensitizing strategy in pancreatic cancer (58, 62). More decisive clinical translation will require randomized trials demonstrating benefit over standard treatment regimens and, ideally, prospectively defined biomarkers capable of identifying tumors dependent on CXCL12–CXCR4-mediated immune exclusion.

Potential biomarkers may include stromal CXCL12 enrichment, the abundance and spatial distribution of CXCR4-expressing immune populations, baseline exclusion of effector T cells from malignant-cell regions, and treatment-induced redistribution of CD8^+^ T cells (29, 63). Longitudinal tumor biopsies combined with spatial profiling and paired assessment of myeloid-cell dynamics will also be important for determining whether CXCR4 blockade converts transient increases in immune-cell accessibility into durable antitumor responses. This issue is particularly relevant because recent clinical evidence indicates that CXCR4 antagonism can increase intratumoral T-cell recruitment while simultaneously enriching immunosuppressive macrophage populations (55).

The CXCL12–CXCR4 axis therefore represents a prototypical stromal immune-exclusion module in cancer. In pancreatic ductal adenocarcinoma, FAP^+^ cancer-associated fibroblasts are a major source of CXCL12, and CXCL12-rich stromal environments can restrict effector T-cell access to malignant-cell regions and diminish the effectiveness of immune checkpoint blockade (29). CXCR4 blockade can increase T-cell mobility and access to tumor-cell compartments and can enhance PD-1/PD-L1-directed therapy in preclinical models and human PDAC tissue systems (29, 63). Early clinical studies further demonstrate that CXCR4-directed combinations can remodel the tumor immune microenvironment, although current evidence remains insufficient to establish CXCR4 blockade as an approved immune-modulatory therapy for solid tumors (58, 62). Importantly, disruption of CXCL12–CXCR4-mediated exclusion does not necessarily eliminate other components of immune resistance. A recent phase II study of plerixafor plus PD-1 inhibition in metastatic PDAC demonstrated increased T-cell recruitment and also enrichment of immunosuppressive macrophages, indicating that CXCR4 blockade may reveal or induce compensatory myeloid suppression (55). Consequently, CXCR4 inhibition is unlikely to achieve maximal efficacy as a stand-alone immune-access strategy. Future approaches should combine improved T-cell accessibility with interventions that enhance T-cell function and restrain suppressive macrophage or other myeloid programs.

Overall, the CXCL12–CXCR4 axis provides a representative example of chemokine-mediated stromal immune exclusion and illustrates why spatial immune remodeling must be evaluated at the ecosystem level rather than solely by changes in lymphocyte abundance. As illustrated in **Figure 2**, CXCL12-rich stromal compartments restrict productive effector T-cell access to tumor-cell regions, whereas CXCR4 blockade can partially restore immune accessibility but may require additional checkpoint- and myeloid-directed interventions to generate durable antitumor immunity (29, 55).

**Figure 2:**
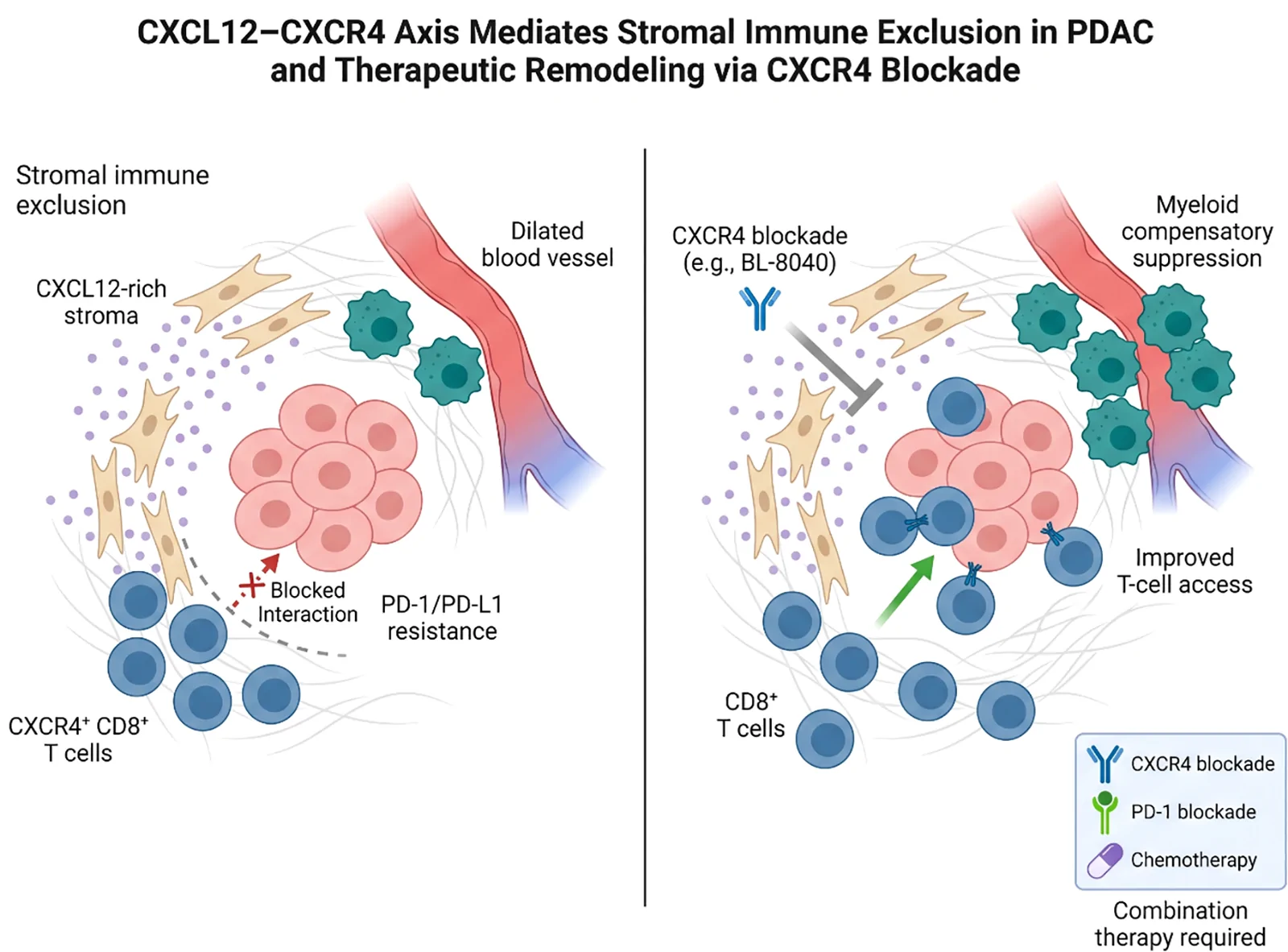
CXCL12–CXCR4-mediated stromal immune exclusion in cancer. CXCL12 produced by CAFs and stromal compartments retains CXCR4^+^ immune cells within the stroma, limiting CD8^+^ T-cell access to tumor-cell nests and reducing responsiveness to PD-1/PD-L1 blockade. CXCR4 antagonism can partially restore immune accessibility, but compensatory macrophage or myeloid accumulation may require rational combination with chemotherapy, checkpoint blockade, or myeloid-targeted therapy.

## 4 CXCR2 ligand axes: neutrophil-dominated immune suppression and therapy resistance

### 4.1 CXCR2 ligands as drivers of neutrophil-rich suppressive niches

The CXCR2 ligand axis represents one of the most important chemokine systems linking inflammation, myeloid-cell recruitment, immune suppression, and therapy resistance in cancer. CXCR2 is a chemokine receptor primarily associated with neutrophil trafficking, although it can also be expressed by granulocytic myeloid-derived suppressor cells, some monocyte/macrophage populations, endothelial cells, and certain tumor cells (64). Its ligands include CXCL1, CXCL2, CXCL3, CXCL5, CXCL6, CXCL7, and CXCL8/IL-8, depending on species and tumor context. In many cancers, these ligands are produced by malignant cells, stromal cells, endothelial cells, macrophages, fibroblasts, or therapy-stressed tumor tissues.

In physiological inflammation, CXCR2 ligands guide neutrophils to sites of infection or tissue damage. In tumors, however, this same program is frequently co-opted to generate neutrophil-rich suppressive niches. These niches are characterized by accumulation of tumor-associated neutrophils, granulocytic MDSCs, inflammatory monocytes, and other suppressive myeloid populations. Rather than supporting productive antitumor immunity, CXCR2^+^ myeloid cells often impair CD8^+^ T-cell infiltration, differentiation, cytotoxicity, and responsiveness to immune checkpoint blockade (65). The immunosuppressive functions of CXCR2-driven myeloid cells are multifaceted. Neutrophils and granulocytic MDSCs can suppress T-cell responses through arginase activity, reactive oxygen species production, inhibitory cytokines, extracellular matrix remodeling, and physical exclusion of lymphocytes from tumor-cell regions. They may also promote angiogenesis, metastasis, epithelial–mesenchymal transition, and tumor-cell survival. In this way, CXCR2 ligand axes link immune-cell trafficking to both spatial organization and functional immune suppression. Importantly, CXCR2 signaling should not be interpreted as a simple “neutrophil recruitment pathway.” Accumulating evidence indicates that CXCR2-associated circuits can influence several layers of tumor biology: they recruit suppressive myeloid cells, reshape local immune niches, inhibit CD8^+^ T-cell function, promote PD-L1 expression, and regulate sensitivity to immunotherapy and chemotherapy. Therefore, CXCR2 ligand axes provide an ideal example of how chemokine–receptor signaling converts inflammatory cues into therapy-resistant immune architectures.

### 4.2 CXCR2 axis in inflammation-associated HCC and immunotherapy resistance

Hepatocellular carcinoma provides a clinically relevant setting in which chronic inflammation, metabolic injury, and myeloid-cell recruitment intersect. This is particularly evident in non-alcoholic steatohepatitis-associated HCC (NASH-HCC), where immune checkpoint blockade may be less effective than in viral-associated HCC in some experimental and clinical datasets (66, 67). Mechanistically, NASH promotes a distinct hepatic immune environment characterized by chronic metabolic inflammation, impaired antitumor immune surveillance, and accumulation or activation of inflammatory immune populations that can compromise productive antitumor responses (66). Neutrophils represent an important component of this myeloid-rich microenvironment and have increasingly been implicated in HCC progression, immune suppression, and therapeutic resistance (68). Within this context, CXCR2 signaling has emerged as a key pathway linking chronic metabolic inflammation and neutrophil recruitment to immunotherapy resistance. In human NASH-HCC, CXCR2-expressing neutrophils are highly represented, and experimental CXCR2 inhibition can reduce neutrophil-mediated immune suppression and restore responsiveness to anti-PD-1 therapy in otherwise ICI-resistant NASH-HCC models (53).

Leslie and colleagues provided important experimental evidence for this concept in *Gut* in 2022 (53). The study was based on the hypothesis that targeting neutrophils through CXCR2 inhibition could sensitize NASH-HCC to immune checkpoint inhibition. The authors characterized neutrophil infiltration in human HCC and mouse models and showed that CXCR2 inhibition reprogrammed the tumor immune microenvironment in a way that promoted responsiveness to immune checkpoint blockade. This work positioned CXCR2-dependent neutrophil recruitment as a mechanistic link between NASH-associated inflammatory liver cancer and poor immunotherapy response. This study is important for the present review because it shows that chemokine–receptor axes can encode disease-etiology-specific immune resistance. In NASH-HCC, resistance to immunotherapy is not merely a property of malignant hepatocytes; rather, it is strongly shaped by the inflammatory tissue context in which tumors arise. CXCR2-dependent neutrophil recruitment and reprogramming contribute to a myeloid-dominated tumor microenvironment that limits productive antitumor immunity. In human NASH-HCC, CXCR2-expressing neutrophils are highly represented, and experimental CXCR2 inhibition can reprogram tumor-associated neutrophils, increase intratumoral dendritic-cell activation and CD8^+^ T-cell accumulation, and restore responsiveness to anti-PD-1 therapy in otherwise ICI-resistant models (53). Thus, the CXCR2 axis provides an important mechanistic link between chronic metabolic injury, neutrophil-rich inflammation, and immune checkpoint resistance. A complementary HCC study by Kwong and colleagues further strengthened the translational relevance of this pathway. Their 2025 *Molecular Therapy* study demonstrated that the myeloid IL-8/CXCR2 circuit contributes to resistance to immune checkpoint blockade in HCC (69). Single-cell RNA sequencing of paired baseline and on-treatment biopsies from patients receiving pembrolizumab showed that IL-8 and CXCR2 were predominantly associated with immunosuppressive myeloid-derived suppressor cells, while elevated circulating IL-8 was associated with poor response to immune checkpoint blockade (69). In an ICB-resistant orthotopic HCC model, pharmacological CXCR2 inhibition with AZD5069 reduced MDSC trafficking and immunosuppressive activity and sensitized tumors to anti-PD-L1 treatment, resulting in reduced tumor growth and prolonged survival (69). Collectively, these studies support a model in which CXCR2-centered myeloid signaling sustains immunotherapy-resistant HCC states and represents a potential target for combination immunotherapy.

These two HCC studies together support a coherent model: CXCR2 ligand signaling recruits and activates suppressive myeloid populations that limit the efficacy of immune checkpoint blockade. In NASH-HCC, this process is linked to chronic metabolic inflammation, whereas in broader HCC contexts, myeloid IL-8/CXCR2 signaling appears to sustain an immunotherapy-resistant microenvironment. These findings suggest that CXCR2 inhibition may be particularly valuable in HCC subsets characterized by neutrophil or myeloid enrichment, high IL-8/CXCR2 activity, and poor T-cell-mediated antitumor immunity. In inflammation-associated HCC, CXCR2-dependent neutrophil recruitment links chronic metabolic injury to immune checkpoint resistance. Leslie et al. demonstrated that CXCR2 inhibition reprograms the neutrophil-rich tumor immune microenvironment and enables immunotherapy responsiveness in NASH-HCC. Consistently, Kwong et al. showed that targeting myeloid IL-8/CXCR2 signaling in HCC reduces suppressive myeloid programs and restores antitumor immune activity. These studies identify the CXCR2 axis as a central myeloid chemokine module that converts inflammatory liver cancer into an immunotherapy-resistant state.

### 4.3 CXCL5–CXCR2 axis in lung cancer: neutrophils, PD-L1, and CD8^+^ T-cell dysfunction

The CXCL5–CXCR2 axis is a representative CXCR2 ligand pathway in lung cancer, where it has been linked to neutrophil accumulation, CD8^+^ T-cell dysfunction, immune checkpoint resistance, and tumor-cell-intrinsic immune evasion. Lung tumors often contain abundant neutrophils, and several studies suggest that these neutrophil-rich environments may limit effective antitumor T-cell immunity. Simoncello and colleagues (28) showed that CXCL5-mediated accumulation of mature neutrophils in lung cancer tissues impairs the differentiation program of anticancer CD8^+^ T cells and limits the efficacy of checkpoint inhibitors. This study is important because it connects a defined chemokine ligand, a specific recruited immune population, and a functional consequence for CD8^+^ T cells. Rather than simply showing that neutrophils are present in lung tumors, the study demonstrated that CXCL5-driven neutrophil accumulation can actively interfere with the generation or maintenance of effective anticancer CD8^+^ T-cell states. This finding supports a key theme of the review: chemokine axes determine not only immune-cell composition but also immune-cell quality. A tumor infiltrated by immune cells may still be functionally immunosuppressed if the dominant recruited population consists of CXCR2^+^ neutrophils rather than cytotoxic lymphocytes. By impairing CD8^+^ T-cell differentiation, CXCL5-driven neutrophil niches can reduce the efficacy of immune checkpoint blockade. This provides a mechanistic explanation for why some tumors with inflammatory infiltrates remain resistant to immunotherapy.

A recent study by Sun and colleagues further suggested that CXCL5 may suppress CD8^+^ T-cell immunity through both tumor-cell-intrinsic and microenvironmental mechanisms. In this study, CXCL5 was reported to upregulate PD-L1 expression in lung cancer cells through phosphorylation of the PXN/AKT pathway while also promoting neutrophil chemotaxis. This mechanism is supported by an earlier lung cancer study showing that CXCL5 can modulate the PD-1/PD-L1 signaling axis, but the specific PXN/AKT-dependent regulation of PD-L1 should currently be interpreted as evidence from one mechanistic study rather than as a broadly established pathway (50, 70). Thus, CXCL5 does not merely recruit suppressive cells; it can also directly enhance immune checkpoint expression on tumor cells. This dual mechanism is conceptually important. It suggests that chemokine ligands can act as multifunctional immune-evasion signals. Within the same tumor ecosystem, CXCL5 can simultaneously promote neutrophil recruitment, impair anticancer CD8^+^ T-cell differentiation, and enhance PD-L1 expression on malignant cells. In lung cancer models, tumor-derived CXCL5 drives the accumulation of mature protumorigenic neutrophils, which suppress the differentiation and cytolytic function of tumor-specific CD8^+^ T cells and contribute to resistance to immune checkpoint blockade (28). These effects converge on reduced cytotoxic T-cell activity and diminished responsiveness to checkpoint inhibition, indicating that the CXCL5–CXCR2 axis functions not only as a trafficking pathway but also as a broader immune-evasion circuit (28). Consistently, recent mechanistic evidence shows that CXCL5 can exert tumor-cell-intrinsic effects in lung cancer. CXCL5 activates CXCR2-dependent phosphorylation of the PXN/AKT signaling pathway, thereby increasing PD-L1 expression in malignant cells, while simultaneously promoting neutrophil chemotaxis (50). Recruited neutrophils can further reinforce this suppressive circuit and aggravate CD8^+^ T-cell dysfunction (50). Thus, the CXCL5–CXCR2 axis illustrates how a single chemokine module can coordinate myeloid recruitment, T-cell dysfunction, and tumor-cell-intrinsic immune evasion within the same tumor microenvironment.

These studies together support a role for CXCL5 in lung cancer immune evasion through neutrophil recruitment and PD-1/PD-L1-associated immune regulation. However, the PXN/AKT-dependent PD-L1 mechanism remains primarily supported by one recent mechanistic study and should be validated in additional lung cancer models and clinical cohorts.

### 4.4 CXCL5 in obesity-associated pancreatic cancer: preliminary evidence for obesity-shaped immune niches

The influence of CXCR2 ligand axes is not limited to tumor-intrinsic factors; it can also be shaped by systemic host conditions. Obesity is increasingly recognized as a modifier of pancreatic cancer risk, inflammatory tone, stromal remodeling, immune composition, and treatment response (71). Experimental and translational studies have shown that obesity can promote adipose tissue inflammation, pancreatic fibrosis/desmoplasia, tumor-associated neutrophil and macrophage accumulation, altered CD8^+^ T-cell/Treg balance, and reduced chemotherapy responsiveness in PDAC (72). In addition, obesity-associated endocrine–exocrine signaling and local pancreatic microenvironmental changes may accelerate oncogenic Kras-driven pancreatic tumorigenesis. Against this broader background, Walsh et al. provided a recent preclinical example linking obesity-associated PDAC to tumor-derived CXCL5, T-cell infiltration, and anti-PD-1 responsiveness (73).

Walsh and colleagues investigated tumor-derived CXCL5 in an obese model of pancreatic cancer and found that depletion of tumor-derived CXCL5 improves T-cell infiltration and anti-PD-1 therapy response. The study showed that CXCL5 depletion *in vivo* was sufficient to promote T-cell infiltration into tumors, and that therapeutic benefit required checkpoint blockade, supporting a model in which CXCL5 limits T-cell access or activity in obesity-associated pancreatic cancer. This study adds an important dimension to the CXCR2 ligand axis (73). It suggests that chemokine-driven immune exclusion is not determined only by tumor genotype or local stromal architecture, but also by systemic metabolic context. Obesity may reinforce CXCL5-associated inflammatory niches, thereby reducing T-cell infiltration and weakening anti-PD-1 efficacy. Conversely, CXCL5 depletion can convert the tumor microenvironment into a more T-cell-permissive state, allowing checkpoint blockade to become more effective. The translational implications of this finding should be interpreted cautiously. At present, direct evidence that CXCL5 defines a clinically actionable obesity-associated immunotherapy-resistance program in human PDAC remains limited. Nevertheless, the Walsh et al. study provides a useful proof-of-concept that host metabolic status may influence chemokine-dependent immune remodeling and checkpoint responsiveness. Future studies should validate whether CXCL5/CXCR2 ligand signatures correlate with body mass index, adipose inflammation, spatial T-cell exclusion, myeloid-cell enrichment, and anti-PD-1 response in human PDAC cohorts. These findings together suggest that obesity-associated inflammation and stromal remodeling may provide a permissive context for chemokine-driven immune suppression in PDAC. However, the specific link between tumor-derived CXCL5, obesity-shaped immune niches, and anti-PD-1 responsiveness is currently supported mainly by preclinical evidence. Therefore, CXCL5 should be presented as a promising but still preliminary candidate mediator of obesity-associated immune remodeling, rather than as an established biomarker or therapeutic target in human PDAC.

### 4.5 CXCR2 and chemotherapy response

Although CXCR2 ligand axes are often discussed in the context of immune checkpoint resistance, they also influence responses to conventional chemotherapy. Chemotherapy can induce inflammatory stress, damage-associated signaling, and chemokine production within tumor tissues. These therapy-induced chemokine programs may recruit suppressive myeloid cells, remodel the tumor microenvironment, and contribute to adaptive resistance. Conversely, blocking chemokine receptors such as CXCR2 may enhance chemotherapy efficacy by reducing suppressive inflammation and directly affecting tumor-cell behavior.

Cheng and colleagues demonstrated that targeting CXCR2 inhibits lung cancer progression and promotes the therapeutic effect of cisplatin. Their study showed that CXCR2 expression was elevated in human lung cancer stroma and tumor cells and was associated with patient prognosis. Inhibition of CXCR2 promoted apoptosis, senescence, EMT-related changes, and anti-proliferative effects in lung cancer cells. *In vivo*, tumor-associated neutrophils infiltrated tumor tissues with upregulated CXCL/CXCR2 signaling and suppressive molecules such as Arg-1 and TGF-β. CXCR2 inhibition reduced these tumor-promoting features and enhanced cisplatin efficacy (74). This study highlights two aspects of CXCR2 biology. First, CXCR2 signaling can act through the tumor microenvironment by regulating tumor-associated neutrophils and suppressive inflammatory mediators. Second, CXCR2 may also act directly on tumor cells, influencing proliferation, survival, senescence, apoptosis, and EMT-related phenotypes (48). Therefore, CXCR2 inhibition may sensitize tumors to chemotherapy through both immune-dependent and tumor-cell-intrinsic mechanisms. This point is important for the therapeutic section of the review. Chemokine-targeted therapy should not be limited to combinations with immune checkpoint blockade. In selected tumors, CXCR2 blockade may enhance chemotherapy by reducing neutrophil-mediated protection, weakening inflammatory survival signals, and increasing tumor-cell susceptibility to cytotoxic stress. This broader therapeutic relevance makes CXCR2 ligand axes central to both immunotherapy resistance and conventional therapy resistance. Beyond immune checkpoint resistance, CXCR2 signaling also contributes to chemotherapy response. In lung cancer, CXCR2 inhibition suppresses tumor progression and enhances cisplatin efficacy by affecting both tumor-associated neutrophil programs and tumor-cell-intrinsic phenotypes such as proliferation, apoptosis, senescence, and EMT (75). These findings indicate that CXCR2-dependent chemokine networks participate in adaptive resistance to conventional cytotoxic therapy as well as immunotherapy.

CXCR2 ligand axes represent a major neutrophil-centered immunosuppressive module in cancer. Through ligands such as CXCL5 and CXCL8/IL-8, tumors and stromal compartments recruit CXCR2^+^ neutrophils, granulocytic MDSCs, and other suppressive myeloid populations, which can impair CD8^+^ T-cell differentiation, reduce cytotoxic function, and limit immune checkpoint efficacy (28, 69). At the same time, CXCR2 ligand signaling can directly regulate tumor-cell phenotypes, including PD-L1 expression, proliferation, survival, EMT, and chemotherapy response (50, 74). The studies discussed in this section support a unified model: CXCR2 ligand axes convert inflammatory chemokine signaling into therapy-resistant immune architecture. In NASH-HCC, CXCR2-dependent neutrophil recruitment links metabolic inflammation to immune checkpoint resistance (53). In HCC more broadly, myeloid IL-8/CXCR2 signaling sustains suppressive myeloid programs (69). In lung cancer, CXCL5 promotes neutrophil accumulation, impairs CD8^+^ T-cell differentiation, and upregulates PD-L1 through PXN/AKT signaling. In obesity-associated pancreatic cancer, tumor-derived CXCL5 limits T-cell infiltration and anti-PD-1 response. In lung cancer chemotherapy models, CXCR2 inhibition enhances cisplatin sensitivity (74). Thus, CXCR2-associated circuits occupy a central position at the intersection of inflammation, immune suppression, spatial niche formation, and treatment resistance. Therapeutically, CXCR2 inhibition may be especially relevant in tumors characterized by neutrophil enrichment, high CXCL5 or IL-8 expression, poor CD8^+^ T-cell infiltration, and resistance to immune checkpoint blockade or chemotherapy (**Figure 3**).

**Figure 3:**
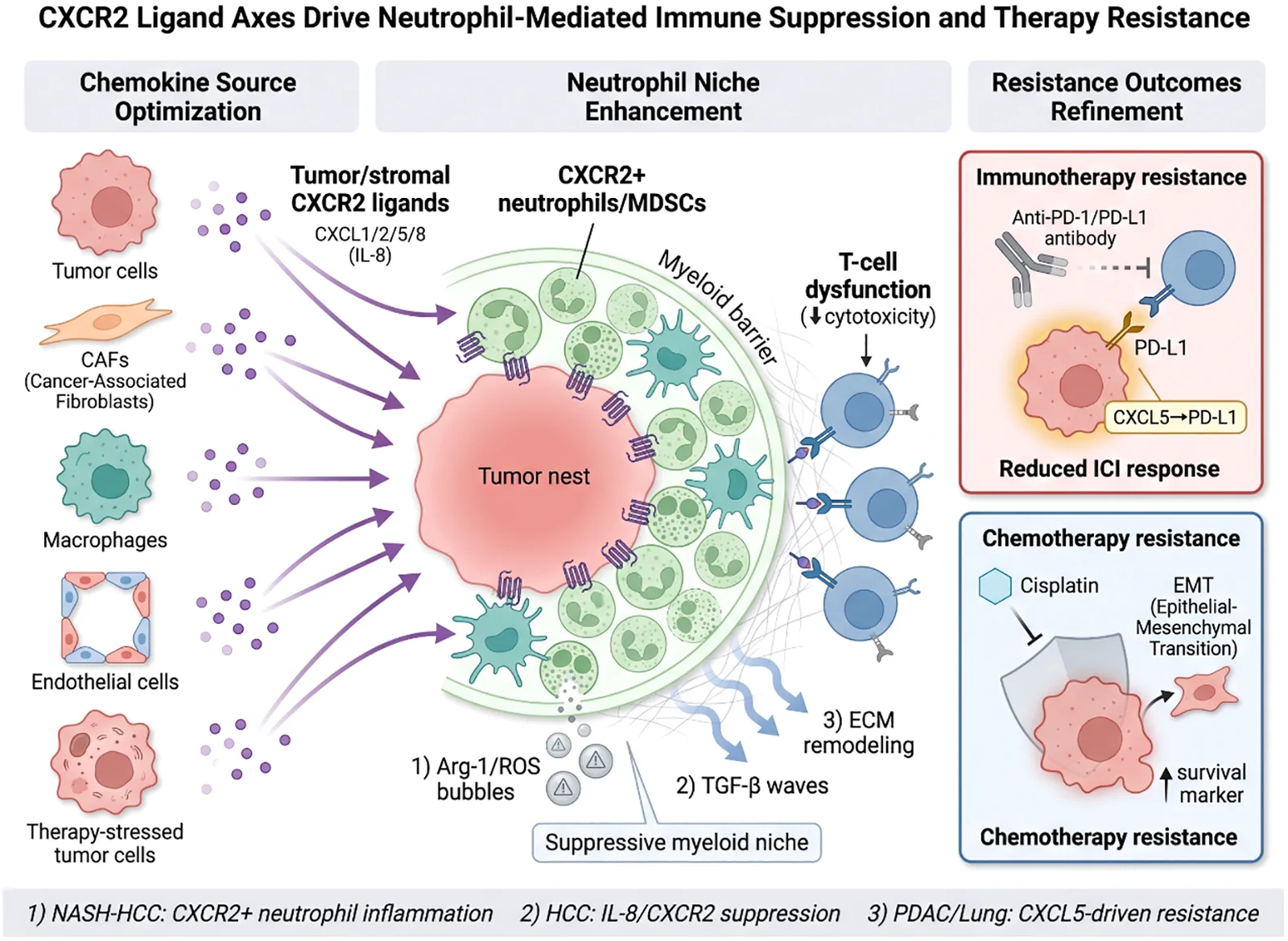
CXCR2 ligand axes in neutrophil-dominated immune suppression and therapy resistance. Tumor, stromal, endothelial, myeloid, and therapy-stressed cells release CXCR2 ligands such as CXCL5 and IL-8, recruiting CXCR2^+^ neutrophils and granulocytic MDSCs. These cells form suppressive myeloid niches that impair CD8^+^ T-cell function, promote PD-L1 expression, and support resistance to immune checkpoint blockade and chemotherapy.

## 5 CCL20–CCR6 axis in Treg, Th17, and B-cell-associated immune remodeling

### 5.1 Biological overview of CCL20–CCR6 signaling

The CCL20–CCR6 axis is a relatively selective chemokine–receptor pathway with important roles in mucosal immunity, inflammatory cell trafficking, lymphocyte positioning, and tumor immune regulation (46). CCL20, also known as macrophage inflammatory protein-3α, is the major ligand for CCR6. Compared with chemokine systems that have multiple ligands and receptors, the CCL20–CCR6 axis is more restricted, which makes it an attractive pathway for studying defined immune-cell recruitment programs in cancer (76). CCR6 is expressed by several immune-cell subsets, including regulatory T cells, Th17 cells, certain memory T-cell subsets, B cells, dendritic cell subsets, and other context-dependent tumor-infiltrating immune populations (77). In the tumor microenvironment, CCL20 can be produced by malignant cells, macrophages, myeloid cells, stromal cells, and inflammatory epithelial compartments (78). This allows CCL20–CCR6 signaling to function as a bridge between tumor cells, myeloid cells, and lymphocyte populations (79).

In cancer, the CCL20–CCR6 axis is frequently associated with Treg recruitment, immune suppression, chronic inflammation, and metastatic niche formation (76). This is particularly important because Tregs are not randomly distributed within tumors. They often accumulate in specific regions where chemokine gradients, antigen exposure, stromal signals, and suppressive cytokines converge. Once recruited, CCR6^+^ Tregs can suppress CD8^+^ T-cell activity, impair dendritic cell function, and reinforce immune tolerance (8). Therefore, CCL20–CCR6 signaling can help establish local immune-suppressive compartments that protect malignant cells from immune elimination. In hepatocellular carcinoma, the CCL20–CCR6 axis mediates selective recruitment of CCR6^+^ Tregs into tumor tissues and is associated with tumor progression and poor prognosis (30). In metastatic prostate cancer, myeloid-derived CCL20 and T-cell CCR6 signaling contribute to an immunosuppressive bone metastatic microenvironment, and disruption of this axis restores T-cell reactivity in preclinical models (46). The biological function of CCL20–CCR6 is context-dependent. In some inflammatory settings, CCR6^+^ T cells may contribute to immune activation or tissue inflammation. However, in many tumors, CCL20–CCR6 signaling favors the accumulation of suppressive lymphocyte populations, especially Tregs, and contributes to immune escape. Importantly, the cellular source of CCL20 may vary across tumor types. In some tumors, malignant cells produce CCL20 directly and recruit CCR6^+^ Tregs (47). In others, macrophages or myeloid cells secrete CCL20 and create a suppressive immune niche. This flexibility makes CCL20–CCR6 a useful example of how the same chemokine axis can be driven by different cellular compartments while converging on similar immunosuppressive outcomes. Within the conceptual framework of this review, the CCL20–CCR6 axis is best understood as a Treg-recruiting and niche-forming module. It does not merely attract immune cells into tumors; it helps determine the local balance between effector and suppressive lymphocytes. By promoting Treg accumulation and shaping metastatic immune microenvironments, this axis contributes to immune evasion and may influence therapeutic response.

### 5.2 CCL20–CCR6 in prostate cancer bone metastasis

Bone metastasis provides a distinct spatial and immunological context for chemokine–receptor signaling. Unlike primary tumors, metastatic lesions develop within a pre-existing tissue niche that contains resident stromal cells, bone marrow immune cells, osteoblasts, osteoclasts, endothelial cells, and hematopoietic elements (80, 81). These cellular components create a complex microenvironment in which tumor cells must adapt to local immune and stromal constraints. Chemokine axes are particularly important in this setting because they help organize immune-cell recruitment and retention within metastatic niches.

A key study by Kfoury and colleagues demonstrated that human prostate cancer bone metastases contain an actionable immunosuppressive microenvironment. By analyzing human prostate cancer bone metastases and relevant models, the study identified an immunosuppressive metastatic niche involving CCL20–CCR6 signaling. CCL20 was enriched in myeloid cells, whereas CCR6 was expressed in T cells. Functional disruption of the CCL20–CCR6 axis restored T-cell reactivity and prolonged survival in preclinical models (46). This study is especially relevant because it links a specific chemokine–receptor axis to immune suppression in a spatially defined metastatic niche. This finding has several implications. First, it shows that CCL20–CCR6 signaling is not restricted to primary tumor immune suppression but can also operate within metastatic microenvironments. Second, it highlights myeloid cells as a source of CCL20, suggesting that metastatic immune suppression can be driven by non-malignant immune compartments rather than tumor cells alone. Third, it demonstrates that disrupting a chemokine axis can restore antitumor T-cell reactivity, indicating that CCL20–CCR6 is not only a biomarker of suppressive niches but also a potential therapeutic vulnerability.

In the context of this review, prostate cancer bone metastasis illustrates how chemokine–receptor signaling contributes to metastatic immune niche formation. The CCL20–CCR6 axis appears to organize communication between myeloid cells and T cells within bone lesions, promoting an immune state that favors tumor persistence. This provides a strong example of the spatial layer of chemokine regulation: CCL20–CCR6 signaling defines where and how suppressive immune interactions occur in metastatic tissue (82). The study also supports a broader principle. Metastatic immune escape may depend not only on tumor-intrinsic adaptations but also on chemokine-guided immune remodeling within the target organ. Therefore, targeting metastatic chemokine niches may represent a complementary strategy to tumor-cell-directed therapy and systemic immunotherapy.

### 5.3 Tumor-derived CCL20 recruits CCR6^+^ Tregs in pancreatic cancer

Pancreatic cancer is characterized by dense stroma, strong immune suppression, limited T-cell infiltration into tumor nests, and poor response to immune checkpoint blockade (83). While CXCL12–CXCR4 signaling is often discussed as a stromal immune-exclusion mechanism in pancreatic cancer, other chemokine axes also contribute to the formation of suppressive immune compartments. Among these, CCL20–CCR6 signaling has emerged as an important pathway linking tumor cells to Treg recruitment.

Tumor-derived CCL20 can directly recruit CCR6^+^ regulatory T cells into pancreatic tumors (47). This mechanism represents a clear example of tumor-to-immune chemokine communication. Instead of passively escaping immune attack, pancreatic cancer cells can actively shape the immune microenvironment by secreting chemokines that attract suppressive lymphocyte populations. Once recruited, CCR6^+^ Tregs may inhibit effector T-cell activation, reduce cytotoxic immune pressure, and reinforce local immune tolerance (8). This mechanism is important because it demonstrates that immune suppression in pancreatic cancer is not mediated only by stromal barriers or myeloid cells. Treg recruitment through CCL20–CCR6 provides an additional suppressive layer. In this setting, the tumor cell itself acts as the source of the chemokine signal, while CCR6-expressing Tregs are the responding immune population. This establishes a direct malignant cell–immune cell communication circuit (83).

The concept also has therapeutic implications. If tumor-derived CCL20 is a major driver of Treg accumulation, then blocking CCL20 production, inhibiting CCR6 signaling, or disrupting downstream Treg function may help reduce immune suppression. Such approaches may be most effective when combined with therapies that promote effector T-cell activity, such as immune checkpoint blockade, CD40 agonists, cancer vaccines, or stromal-modulating strategies. Within the broader framework of chemokine-mediated immune exclusion, tumor-derived CCL20 differs from CXCL12-mediated stromal exclusion. CXCL12–CXCR4 mainly restricts immune-cell access to tumor nests, whereas CCL20–CCR6 actively recruits suppressive lymphocytes into the tumor microenvironment (84). Both mechanisms reduce effective antitumor immunity, but they do so through different spatial and cellular routes. They together help explain why pancreatic cancer remains highly resistant to immunotherapy.

### 5.4 TAM-derived CCL20 and Treg recruitment in HCC

The CCL20–CCR6 axis can also be driven by tumor-associated macrophages. This macrophage-derived route is especially important because TAMs are major regulators of immune suppression, angiogenesis, matrix remodeling, tumor-cell survival, and therapeutic resistance (85, 86). In many tumors, TAMs do not simply respond to chemokine signals; they produce chemokines that organize local immune-cell composition and function.

In hepatocellular carcinoma, galectin-1-induced TAMs have been shown to repress antitumor immunity through Treg recruitment (87). Galectin-1 promotes an immunosuppressive TAM phenotype, and these TAMs increase CCL20 secretion. CCL20 then recruits Tregs into the tumor microenvironment, reinforcing immune suppression. Targeting galectin-1 reduces CCL20 production, decreases Treg recruitment, and improves antitumor immune activity. This study expands the CCL20–CCR6 model in two important ways. First, it shows that CCL20-driven Treg recruitment is not limited to tumor-cell-derived chemokine production. Macrophages can act as the chemokine source and thereby establish a macrophage-instructed suppressive lymphocyte niche. Second, it positions galectin-1 upstream of CCL20 secretion, suggesting that chemokine circuits may be regulated by broader immunosuppressive programs within the tumor microenvironment.

The macrophage-derived CCL20 model also emphasizes the reciprocal nature of immune–tumor communication. Tumor cells and stromal signals may educate TAMs into an immunosuppressive phenotype. These TAMs then produce CCL20, recruit Tregs, and further suppress antitumor immunity (88). Thus, immune suppression emerges from a multicellular circuit rather than a single ligand–receptor pair (89). This is highly relevant to the theme of this review because chemokine axes often operate as components of larger spatial and functional networks. From a therapeutic perspective, this finding suggests that targeting upstream regulators such as galectin-1 may indirectly disrupt CCL20-mediated Treg recruitment. Alternatively, direct blockade of CCL20–CCR6 signaling may reduce Treg accumulation in macrophage-rich tumors. However, because macrophages can produce multiple chemokines and suppressive mediators, effective strategies may need to combine chemokine-axis blockade with TAM reprogramming or immune checkpoint inhibition.

The CCL20–CCR6 axis represents a flexible and context-dependent chemokine module that frequently promotes Treg recruitment, immune suppression, and metastatic niche formation. CCL20 can be produced by tumor cells, myeloid cells, or tumor-associated macrophages, whereas CCR6 is expressed by Tregs and other immune-cell subsets. This allows CCL20–CCR6 signaling to connect malignant cells, myeloid populations, and suppressive lymphocytes within the tumor microenvironment. In prostate cancer bone metastasis, CCL20–CCR6 signaling contributes to an actionable immunosuppressive metastatic niche, and disruption of this axis restores T-cell reactivity. In pancreatic cancer, tumor-derived CCL20 recruits CCR6^+^ Tregs and establishes a Treg-rich suppressive compartment. In HCC, galectin-1-induced TAMs secrete CCL20 to recruit Tregs, demonstrating that macrophages can also drive this axis. Overall, CCL20–CCR6 signaling should be viewed as a Treg-recruiting and niche-forming axis rather than a simple lymphocyte trafficking pathway. Its importance is particularly evident in tumors where immune suppression is organized through Treg accumulation, myeloid–lymphocyte communication, or metastatic microenvironment remodeling. Targeting this axis may help weaken local immune tolerance and enhance the efficacy of immunotherapy, especially when combined with approaches that activate effector T cells or reprogram suppressive macrophages. The CCL20–CCR6 axis functions as a flexible Treg-recruiting module driven by tumor cells, myeloid cells, or TAMs. As illustrated in **Figure 4**, this axis creates Treg-rich suppressive niches in primary and metastatic tumors, limiting CD8^+^ T-cell activity and promoting immune escape.

**Figure 4:**
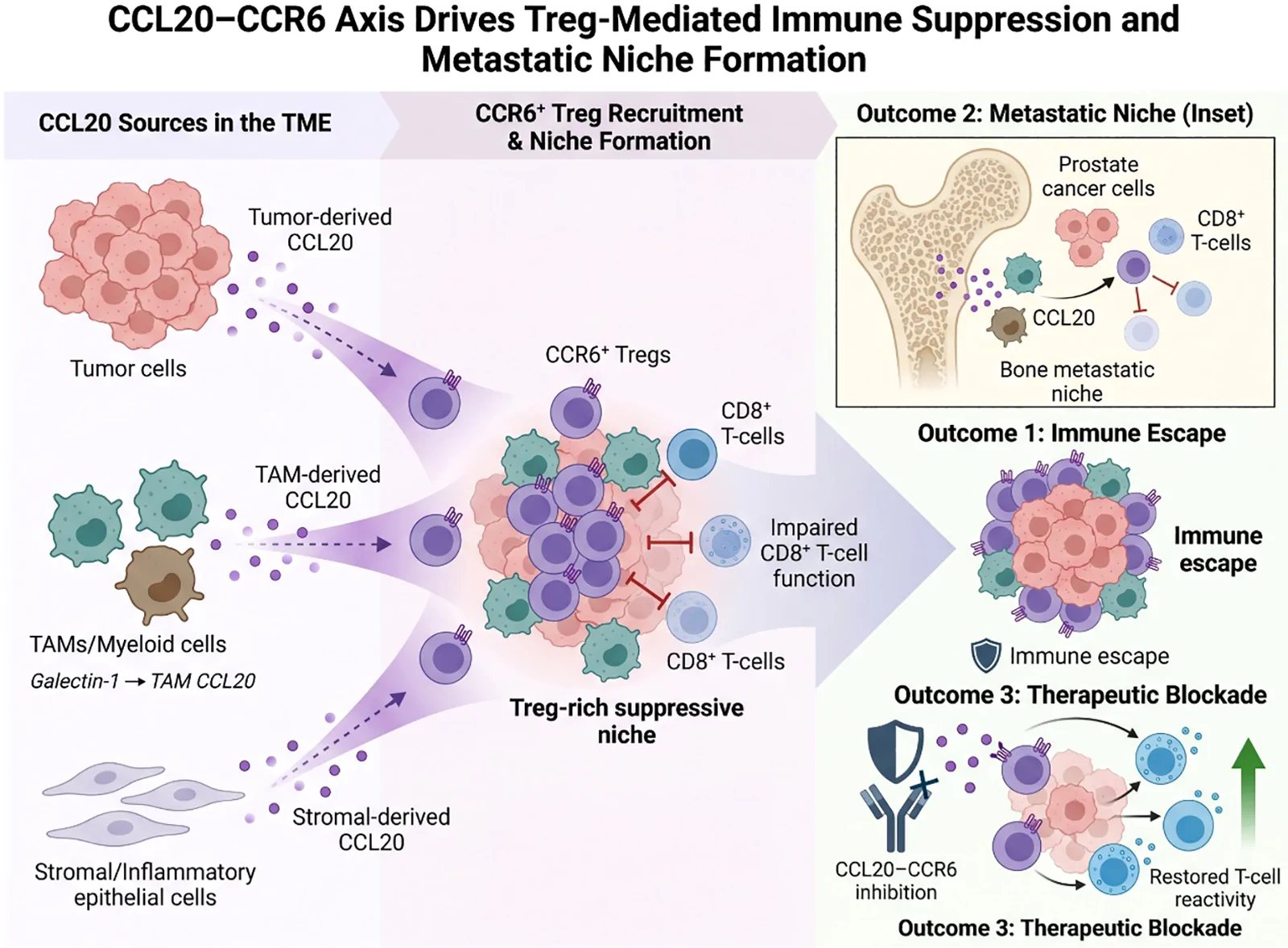
CCL20–CCR6-mediated Treg recruitment and suppressive niche formation. CCL20 produced by tumor cells, myeloid cells, or TAMs recruits CCR6^+^ Tregs into primary or metastatic tumor microenvironments. These Treg-rich niches suppress CD8^+^ T-cell activity and promote immune escape. In bone metastatic prostate cancer, myeloid-derived CCL20 contributes to an actionable suppressive niche. Blocking CCL20–CCR6 signaling may reduce Treg recruitment and restore T-cell reactivity.

### 5.5 Beyond Tregs: Th17 and B-cell involvement in CCL20–CCR6 niches

Beyond Treg recruitment, the CCL20–CCR6 axis also shapes Th17- and B-cell-associated tumor immune niches. CCR6 is highly expressed by Th17 cells and represents an important chemokine receptor controlling their migration toward CCL20-enriched inflammatory tissues and tumor microenvironments (76, 90). In cervical cancer, tumor-infiltrating Th17 cells exhibit increased CCR6 expression and are preferentially recruited through the CCL20–CCR6 pathway, supporting the role of this axis in organizing IL-17-associated inflammatory compartments within tumors (91). Consistently, cervical cancer-associated fibroblasts can be instructed by tumor-derived IL-6 to produce CCL20 through C/EBPβ-dependent signaling, thereby promoting CCR6-dependent recruitment of Th17 cells (92). Evidence from oral squamous cell carcinoma further supports the involvement of CCR6-associated CD4^+^ T-cell programs in the tumor microenvironment, where increased frequencies of CD45RO^+^CCR6^+^CD4^+^ and IL-17^+^ cells have been observed in selected patient populations (93). Importantly, Th17 cells have context-dependent functions in cancer. Depending on tumor type and local cytokine signals, Th17/IL-17-associated responses may promote chronic inflammation, angiogenesis, recruitment of suppressive myeloid populations, and tumor progression, whereas in other settings Th17 cells may acquire inflammatory or Th1-like properties that support effector immunity (90). Therefore, CCL20–CCR6-driven Th17 recruitment should be interpreted according to tumor type, cytokine context, cellular plasticity, and the composition of accompanying immune populations. B cells may also participate in CCL20–CCR6-mediated immune remodeling. CCR6 is expressed by several activated and memory B-cell populations, and the CCR6–CCL20 axis contributes to B-cell positioning and the organization of humoral immune responses (94). Its relevance extends to cancer. In hepatocellular carcinoma, tumor cell-derived CCL20 was shown to recruit CCR6-high CD19^+^CD5^+^ B cells. These infiltrating B cells promoted tumor progression and were associated with enhanced angiogenic activity, whereas CCL20 neutralization or inhibition of B-cell infiltration attenuated tumor growth in experimental models (95). Thus, CCL20–CCR6 signaling can coordinate not only Treg recruitment but also Th17- and B-cell-associated immune remodeling. Collectively, these findings broaden the interpretation of the CCL20–CCR6 axis beyond a Treg-centered model. Rather than acting on a single suppressive lymphocyte population, CCL20 may engage multiple CCR6^+^ immune subsets, including Tregs, Th17 cells, and B cells, whose biological consequences are determined by tumor type, tissue context, cellular state, and the surrounding cytokine and chemokine network (76).

## 6 CCL5–CCR5 axis: perivascular macrophage niches, immune remodeling, and adaptive resistance

### 6.1 Context-dependent role of CCL5–CCR5 signaling

Compared with the CXCR2 ligand–CXCR2 and CCL20–CCR6 axes, the CCL5–CCR5 axis is more context dependent because CCR5 can be expressed by multiple immune and malignant cell populations, including effector lymphocytes, suppressive myeloid cells, macrophage subsets, Tregs, and tumor cells. The CCL5–CCR5 axis is a highly context-dependent chemokine pathway involved in immune-cell recruitment, inflammatory responses, tumor progression, and therapeutic resistance (96). CCL5, also known as RANTES, can be produced by tumor cells, T cells, macrophages, stromal cells, endothelial cells, and therapy-stressed tissues. Its receptor CCR5 is expressed by multiple immune-cell populations, including T cells, macrophages, regulatory T cells, monocytes, and some myeloid subsets. In certain tumor contexts, CCR5 may also be expressed by malignant cells, allowing CCL5–CCR5 signaling to directly influence tumor-cell behavior. Unlike some chemokine axes that are more consistently associated with a single immune-cell population, CCL5–CCR5 signaling can produce different biological outcomes depending on tumor type, inflammatory state, receptor-expressing cell type, and spatial context. In some inflammatory settings, CCL5–CCR5 may contribute to effector T-cell recruitment and immune activation. However, in many cancers, this axis is associated with recruitment or retention of immunosuppressive immune populations, including tumor-associated macrophages and regulatory T cells. It may also promote metastasis, angiogenesis, epithelial–mesenchymal transition, and resistance to therapy (97). This dual nature makes CCL5–CCR5 particularly important for a review focused on immune–tumor communication. The axis cannot be interpreted simply as “pro-tumor” or “anti-tumor.” Its effect depends on where the signal is produced, which cells express CCR5, and what functional state those cells acquire after responding to CCL5—for example, CCL5 produced in an inflamed tumor region may recruit T cells under some conditions, whereas CCL5 produced within a macrophage-rich or stromal niche may reinforce immune suppression. Similarly, CCR5 expression on immune cells may regulate cell trafficking and retention, while CCR5 expression on tumor cells may support survival and therapy adaptation. Therefore, the CCL5–CCR5 axis is best understood as a context-sensitive immune remodeling module. It can alter immune-cell composition, organize spatial immune niches, and regulate tumor-cell responses to therapeutic stress. This complexity is well illustrated by three experimental settings: BAP1-mutant clear cell renal cell carcinoma, CCR5-dependent perivascular macrophage niches in breast cancer, and CCL5–CCR5–CYP1A1-mediated therapy resistance in liver cancer.

### 6.2 CCR5 blockade in BAP1-mutant clear cell renal cell carcinoma

The therapeutic relevance of CCL5–CCR5 signaling is strongly influenced by tumor genetic context. In clear cell renal cell carcinoma, loss-of-function mutations in BAP1 are associated with aggressive disease and distinct immune features (98, 99). A study by Zhou and colleagues showed that CCR5 blockade inflames antitumor immunity in BAP1-mutant clear cell renal cell carcinoma (100). This work linked a defined tumor genetic background to a chemokine-receptor dependency within the immune microenvironment.

In BAP1-mutant clear cell renal cell carcinoma, CCR5 signaling contributes to an immunosuppressive tumor microenvironment. Blocking CCR5 enhances antitumor immunity, suggesting that the CCL5–CCR5 axis helps maintain immune suppression in this genetic subtype. This finding is important because it suggests that chemokine-targeted therapy may be most effective when guided by tumor-intrinsic molecular features rather than applied uniformly across all patients. This study also provides a useful example of how chemokine axes connect tumor genetics with immune ecology. BAP1 mutation may shape cytokine and chemokine programs that influence immune-cell recruitment, activation, or suppression. CCR5 blockade can then disrupt these programs and shift the tumor microenvironment toward a more inflamed state. In this sense, CCL5–CCR5 signaling acts as a bridge between tumor genotype and immune phenotype. For the current review, this example supports two important principles. First, chemokine–receptor axes may define vulnerabilities in molecularly selected tumor subsets. Second, targeting chemokine receptors may not simply block cell migration; it can reprogram the immune state of the tumor. In BAP1-mutant clear cell renal cell carcinoma, CCR5 blockade appears to convert a more suppressive immune environment into one that is more permissive for antitumor immunity. The broader implication is that future chemokine-targeted strategies should incorporate biomarkers of tumor genotype, immune-cell composition, chemokine expression, and spatial organization (101). Such biomarker-driven approaches may help identify patients most likely to benefit from CCR5 blockade or other chemokine-directed interventions.

### 6.3 CCR5-dependent perivascular macrophage niche in breast cancer

The CCL5–CCR5 axis is also important for spatial niche formation. A particularly informative example comes from breast cancer, where LYVE-1^+^ macrophages have been shown to form a collaborative CCR5-dependent perivascular niche. This finding is highly relevant to the spatial focus of this review because it demonstrates that chemokine–receptor signaling can organize anatomically defined immune structures rather than merely recruit immune cells into tumors. Perivascular niches are specialized microenvironments located around blood vessels. They are important because blood vessels serve as entry points for immune cells, routes for tumor-cell dissemination, and sites of interaction between endothelial cells, macrophages, lymphocytes, and stromal components (102). Macrophages positioned around vessels can regulate vascular permeability, immune-cell extravasation, matrix remodeling, and therapy response. Therefore, a macrophage-rich perivascular niche can strongly influence whether effector immune cells successfully enter tumor tissue and whether chemotherapy reaches tumor cells effectively.

In murine breast cancer, LYVE-1^+^ macrophages form a CCR5-dependent perivascular niche that influences CD8^+^ T-cell recruitment and chemotherapy response (31). This observation expands the functional meaning of chemokine signaling. CCR5 does not only guide macrophages into tumors; it helps maintain a spatially organized macrophage compartment with direct consequences for immune-cell access and treatment outcome. The niche is “collaborative” because its function depends on interactions among macrophages, vascular cells, T cells, and tumor cells within a defined anatomical region. This spatial mechanism is distinct from diffuse chemokine-mediated recruitment. In diffuse recruitment, chemokines broadly increase the number of immune cells within a tumor. In niche formation, chemokines help position cells in specific microanatomical compartments where they acquire specialized functions. The CCR5-dependent perivascular macrophage niche illustrates this second mode of chemokine action. LYVE-1^+^ tumor-associated macrophages form coordinated multicellular structures proximal to tumor vasculature, and CCR5-dependent signaling is required for their organization; disruption of these structures increases CD8^+^ T-cell recruitment and enhances chemotherapy responses (31). It shows that tumor immunity is governed not only by immune-cell abundance but also by immune-cell geography. This study is particularly useful for the theme of spatial niches. It supports the idea that chemokine–receptor axes establish cellular neighborhoods that shape therapeutic response. In breast cancer, perivascular macrophage populations have also been implicated in limiting chemotherapy efficacy and promoting tumor regrowth (103). Thus, targeting CCR5 may alter the structure and function of perivascular immune niches, potentially improving the delivery and efficacy of anticancer therapies.

### 6.4 CCL5–CCR5–CYP1A1 pathway in liver cancer therapy resistance

In addition to regulating immune-cell recruitment and spatial niche formation, the CCL5–CCR5 axis can directly support tumor-cell survival under therapeutic pressure. This tumor-cell-intrinsic function is particularly important for understanding adaptive resistance to combination therapies (104, 105). In liver cancer, preliminary evidence from one recent mechanistic study suggests that the CCL5–CCR5–CYP1A1 pathway may promote tumor-cell survival during combined targeted and immune-based therapy. Wang and colleagues identified this pathway through RNA-seq analysis of CCL5-stimulated Huh7 cells and experimentally validated that CCL5–CCR5–CYP1A1 signaling can mediate resistance of liver cancer cells to lenvatinib. Because CYP1A1 participates in xenobiotic metabolism and stress adaptation, this finding raises the possibility that CCL5–CCR5 signaling may help liver cancer cells tolerate therapeutic pressure by engaging metabolic or detoxification-related programs. However, the CYP1A1-specific mechanism should be regarded as preliminary until it is confirmed in independent models, patient-derived systems, and clinically annotated liver cancer samples (32). This finding broadens the role of CCL5–CCR5 beyond immune-cell trafficking. While many chemokine studies focus on immune recruitment, the liver cancer model demonstrates that chemokine receptors expressed by tumor cells can directly regulate survival programs. Under therapy pressure, tumor cells may exploit CCL5–CCR5 signaling to activate downstream adaptive pathways, thereby reducing the effectiveness of targeted therapy and immunotherapy combinations. The concept of adaptive resistance is central here. Cancer therapies can reshape the tumor microenvironment and induce compensatory signaling programs. Chemokines produced by tumor cells, immune cells, or stromal compartments may create feedback loops that support tumor-cell persistence. In this setting, CCL5–CCR5 signaling functions not only as an immune communication axis but also as a survival pathway that helps malignant cells adapt to treatment. This mechanism has therapeutic implications. Blocking CCR5 may not only modulate immune-cell recruitment but also weaken tumor-cell-intrinsic survival programs. In liver cancer, combining CCR5-targeted therapy with immune checkpoint blockade or targeted therapy may therefore help overcome resistance driven by CCL5–CCR5–CYP1A1 signaling. However, because CCL5–CCR5 can have different functions in different immune compartments, patient selection and mechanistic validation will be essential.

The CCL5–CCR5 axis is a context-dependent chemokine module that links immune remodeling, spatial niche formation, and adaptive therapy resistance. Its biological effects depend on tumor type, genetic background, cellular source of CCL5, receptor-expressing population, and tissue localization. In BAP1-mutant clear cell renal cell carcinoma, CCR5 signaling contributes to an immunosuppressive tumor microenvironment, and CCR5 blockade can enhance antitumor immunity. This example suggests that tumor genetic context may define vulnerability to chemokine-receptor targeting. In breast cancer, CCR5-dependent signaling organizes LYVE-1^+^ macrophages into a perivascular niche that influences CD8^+^ T-cell recruitment and chemotherapy response, illustrating how chemokine axes can create anatomically defined immune compartments. In liver cancer, CCL5–CCR5 signaling directly supports tumor-cell survival under combined targeted and immune therapeutic pressure through the CCL5–CCR5–CYP1A1 pathway. These studies together show that CCL5–CCR5 signaling should not be viewed simply as a leukocyte recruitment pathway. It instead functions as a flexible communication axis that can remodel immune states, organize spatial macrophage niches, and promote tumor-cell adaptation during therapy (106). This context dependency also creates a therapeutic challenge: CCR5 blockade may be beneficial in selected tumors, but its effects will likely depend on immune composition, spatial niche structure, and tumor-cell dependence on CCR5-associated survival programs. The CCL5–CCR5 axis should be interpreted as a context-dependent immune remodeling module rather than a uniformly pro- or anti-tumor pathway. As illustrated in **Figure 5**, this axis can organize perivascular macrophage niches and directly support tumor-cell adaptive resistance under therapeutic pressure.

**Figure 5:**
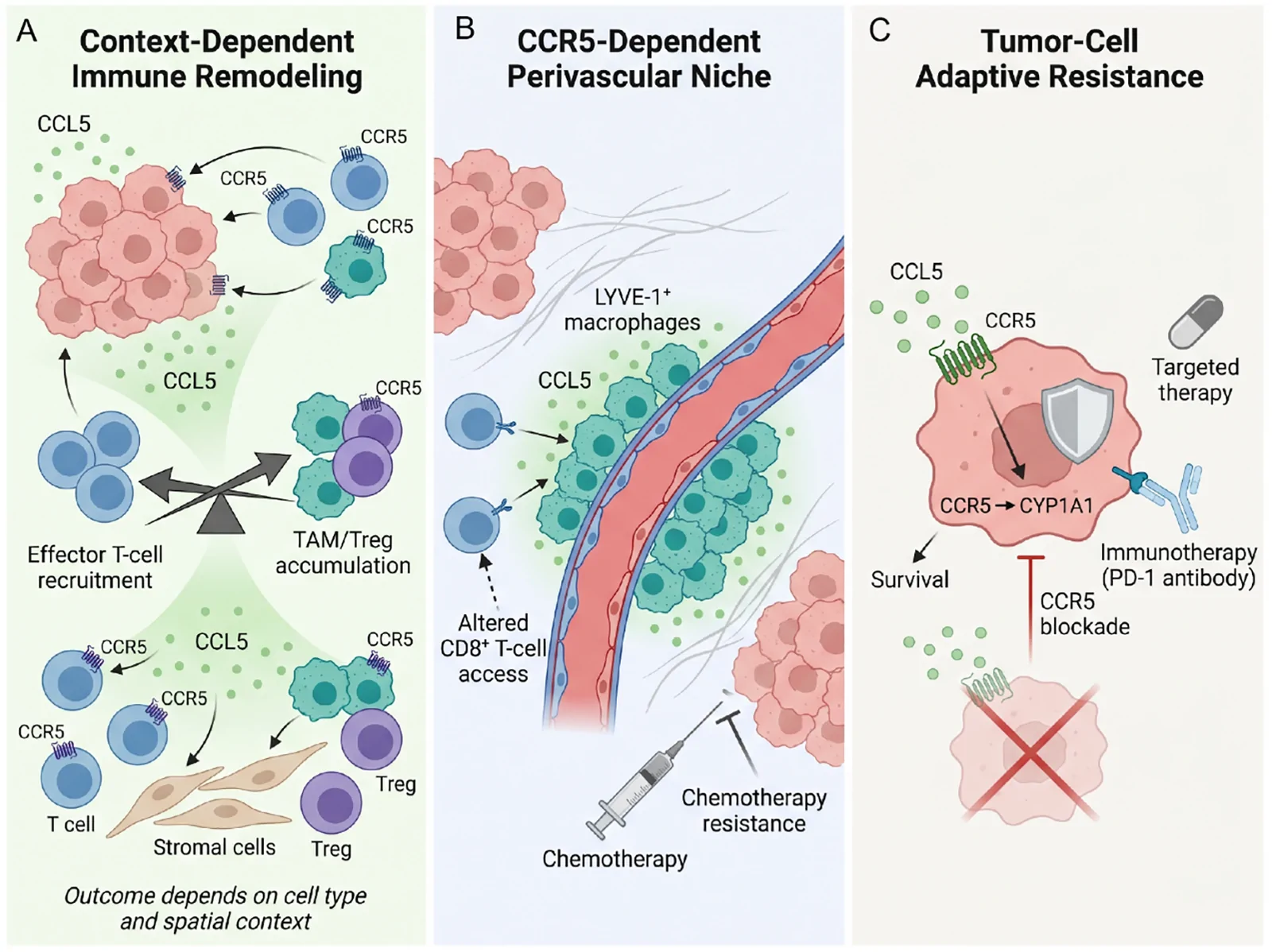
Context-dependent CCL5–CCR5 signaling in immune remodeling and therapy resistance. **(A)** CCL5–CCR5 outcomes depend on receptor-expressing cell type and spatial context, balancing effector T-cell recruitment with TAM/Treg accumulation. **(B)** CCR5-dependent LYVE-1^+^ perivascular macrophage niches may alter CD8^+^ T-cell access and contribute to chemotherapy resistance. **(C)** Preliminary evidence suggests that tumor-cell CCL5–CCR5 signaling can activate CYP1A1-associated adaptive survival, while CCR5 blockade may improve responses to targeted therapy and PD-1 blockade.

### 6.5 Context-dependent outcomes of CCL5–CCR5 signaling

These examples indicate that CCL5–CCR5 signaling should not be interpreted as a uniformly pro-tumorigenic or anti-tumorigenic pathway. Its biological consequence depends on at least three variables: the dominant CCR5-expressing cell type, the spatial niche in which signaling occurs, and the treatment or genetic context that shapes downstream responses. When CCR5 signaling is enriched in suppressive myeloid cells, tumor-associated macrophages, Tregs, or perivascular macrophage niches, CCL5–CCR5 tends to support immune suppression, impaired CD8^+^ T-cell recruitment, and therapy resistance (31, 107). When CCR5 signaling acts directly on malignant cells under therapeutic pressure, it may promote adaptive survival programs, as suggested by the CCL5–CCR5–CYP1A1 pathway in liver cancer (32). By contrast, in inflamed tumors where CCR5^+^ effector T cells or NK cells dominate, CCL5–CCR5 signaling may theoretically support antitumor immune recruitment rather than immune evasion (108, 109). Therefore, the expected outcome of CCL5–CCR5 signaling is best predicted by cellular source, receptor-bearing target cells, spatial localization, and treatment context rather than by ligand or receptor expression alone.

### 6.6 T-cell-associated roles of the CCL5–CCR5 axis

The CCL5–CCR5 axis also has important T-cell-associated functions that should be distinguished from its effects on macrophages or malignant cells. CCR5 can be expressed by activated CD8^+^ T cells, CD4^+^ effector T cells, memory-like T cells, and regulatory T cells, meaning that CCL5 may either support antitumor lymphocyte recruitment or reinforce immune suppression depending on the dominant CCR5^+^ T-cell population (110). In immune-inflamed tumors, CCL5 may contribute to recruitment or positioning of effector T cells, thereby supporting local antitumor immunity—for example, CD40-driven antitumor immunity in pancreatic cancer models was linked to CCL5-mediated CD4^+^ T-cell tumor infiltration, and recent single-cell studies suggest that CCR5 expression can define functionally distinct intratumoral CD8^+^ T-cell states. However, the same axis can also promote Treg-dependent immune escape. In colorectal cancer, tumor-derived CCL5 was shown to recruit CCR5^+^ Tregs and enhance TGF-β-mediated killing of CD8^+^ T cells, thereby suppressing antitumor immunity (111). These findings indicate that CCL5–CCR5 signaling should not be interpreted simply as either beneficial or detrimental for T-cell immunity. Its outcome depends on whether CCL5 primarily attracts effector CD8^+^/CD4^+^ T cells, Tregs, or mixed T-cell states, as well as on the accompanying cytokine context, antigen presentation, myeloid composition, and spatial localization within the tumor microenvironment (112).

## 7 CCL2-associated macrophage circuits: monocyte recruitment, TAM polarization, and immunotherapy sensitization

### 7.1 CCL2/CCR2 axis and macrophage-dominated immune suppression

The CCL2/CCR2 axis is one of the most extensively studied chemokine pathways involved in monocyte recruitment and macrophage accumulation in cancer. CCL2, also known as monocyte chemoattractant protein-1, can be secreted by tumor cells, stromal cells, endothelial cells, fibroblasts, and immune cells within the tumor microenvironment. Its major receptor, CCR2, is highly expressed on inflammatory monocytes and monocyte-derived macrophage precursors (113). Through this ligand–receptor interaction, CCL2 promotes the trafficking of CCR2^+^ monocytes from the bone marrow and circulation into tumor tissues (114). After entering the tumor microenvironment, recruited monocytes can differentiate into tumor-associated macrophages. These TAMs are functionally heterogeneous. In many tumors, they acquire immunosuppressive, pro-angiogenic, pro-metastatic, and tissue-remodeling phenotypes (115). They may suppress CD8^+^ T-cell activation, reduce antigen presentation, secrete inhibitory cytokines, promote extracellular matrix remodeling, support tumor-cell invasion, and facilitate resistance to therapy (116). In this way, CCL2-associated signaling can convert monocyte recruitment into macrophage-dominated immune suppression (117).

However, the CCL2/CCR2 axis should be interpreted cautiously. Not all monocytes and macrophages are uniformly tumor-promoting. Their functions depend on tumor type, disease stage, local cytokine signals, metabolic conditions, spatial location, and treatment context (118). Some macrophage populations may support antigen presentation, tissue inflammation, or antitumor immunity under specific conditions (119). Therefore, CCL2-driven monocyte recruitment does not automatically imply immune suppression. The biological outcome depends on how recruited monocytes are educated after entering the tumor microenvironment. This context dependency is important for therapeutic development. Blocking CCL2 or CCR2 may reduce suppressive TAM accumulation in macrophage-rich tumors, but it may also affect inflammatory or antigen-presenting myeloid subsets. Conversely, strategies that do not simply deplete macrophages but instead repolarize them toward antitumor phenotypes may provide a more nuanced approach (120). In this sense, the CCL2 axis is not only a recruitment pathway but also part of a broader macrophage-state regulatory network. Within the framework of this review, CCL2-associated circuits represent a key example of chemokine-mediated immune–tumor communication. They determine the influx of monocyte precursors, contribute to macrophage-rich spatial niches, and influence whether the tumor microenvironment supports immune suppression or immunotherapy responsiveness. This is especially relevant for tumors such as hepatocellular carcinoma, pancreatic cancer, breast cancer, and colorectal cancer, where macrophage accumulation is often associated with poor prognosis and therapeutic resistance (117, 121–123).

### 7.2 SLAMF7-regulated CCL2 signaling in HCC

Hepatocellular carcinoma is commonly characterized by chronic inflammation, myeloid-cell infiltration, immune exhaustion, and variable responsiveness to immune checkpoint blockade. In this context, macrophage-associated chemokine circuits are particularly important because they can determine whether the tumor microenvironment remains immunosuppressive or becomes permissive to antitumor immunity. Weng and colleagues identified SLAMF7-regulated CCL2 signaling as a mechanism that maintains immunosuppressive macrophage states in HCC (54). Their study showed that this pathway contributes to the persistence of macrophage-mediated immune suppression. Targeting SLAMF7-regulated CCL2 signaling repolarized immunosuppressive macrophages and sensitized HCC to immunotherapy. This finding is important because it moves beyond the simple idea of blocking monocyte recruitment and instead highlights a strategy for macrophage reprogramming. This study illustrates several key principles. First, CCL2-associated signaling can regulate macrophage state, not only macrophage abundance. The therapeutic effect was linked to repolarization of immunosuppressive macrophages, suggesting that the functional phenotype of TAMs is as important as their number. Second, CCL2 signaling may operate downstream of broader immune-regulatory molecules such as SLAMF7, indicating that chemokine pathways are embedded within larger immune signaling networks. Third, targeting this circuit can improve immunotherapy response, supporting the idea that macrophage-associated chemokine axes are actionable resistance mechanisms (54). For the present review, this HCC study provides a useful example of how CCL2-associated circuits connect monocyte/macrophage biology with immunotherapy sensitization. In macrophage-rich tumors, immune checkpoint blockade may fail not because T cells are completely absent, but because suppressive macrophages prevent productive antitumor immunity. Reprogramming the CCL2-associated macrophage circuit can reduce this suppressive pressure and make tumors more responsive to immune-based therapy (117). This mechanism also complements the CXCR2 and CCL20–CCR6 axes discussed above. Whereas CXCR2 ligand axes often center on neutrophil-rich suppression and CCL20–CCR6 frequently promotes Treg recruitment, CCL2-associated signaling mainly shapes the monocyte–macrophage compartment (28, 30). These chemokine systems together define distinct but interconnected suppressive immune modules within the tumor microenvironment.

CCL2-associated signaling links monocyte trafficking, TAM polarization, immune suppression, and immunotherapy response. By recruiting CCR2^+^ monocytes and supporting their differentiation into TAMs, the CCL2/CCR2 axis can promote macrophage-dominated tumor microenvironments that suppress T-cell function, support angiogenesis, facilitate metastasis, and reduce therapeutic efficacy (124). At the same time, this axis is highly context-dependent. Recruited monocytes may acquire different macrophage states depending on local cues, spatial niches, and treatment conditions (125). Therefore, therapeutic strategies should not be limited to simple macrophage depletion or chemokine blockade. More precise approaches may involve disrupting suppressive CCL2-associated circuits while preserving or inducing macrophage functions that support antigen presentation and antitumor immunity (126). The study of SLAMF7-regulated CCL2 signaling in HCC demonstrates this principle. Targeting CCL2/CCR2 signaling within this pathway repolarizes immunosuppressive macrophages and sensitizes tumors to immunotherapy, supporting the therapeutic potential of macrophage-associated chemokine circuits (54). Overall, CCL2-associated signaling is especially relevant in macrophage-rich tumors where TAM-mediated suppression represents a major barrier to effective immune checkpoint blockade. CCL2-associated signaling links monocyte recruitment with TAM polarization and macrophage-dominated immune suppression. As illustrated in **Figure 6**, targeting the SLAMF7–CCL2 macrophage circuit may repolarize suppressive TAMs and sensitize tumors to immune checkpoint blockade.

**Figure 6:**
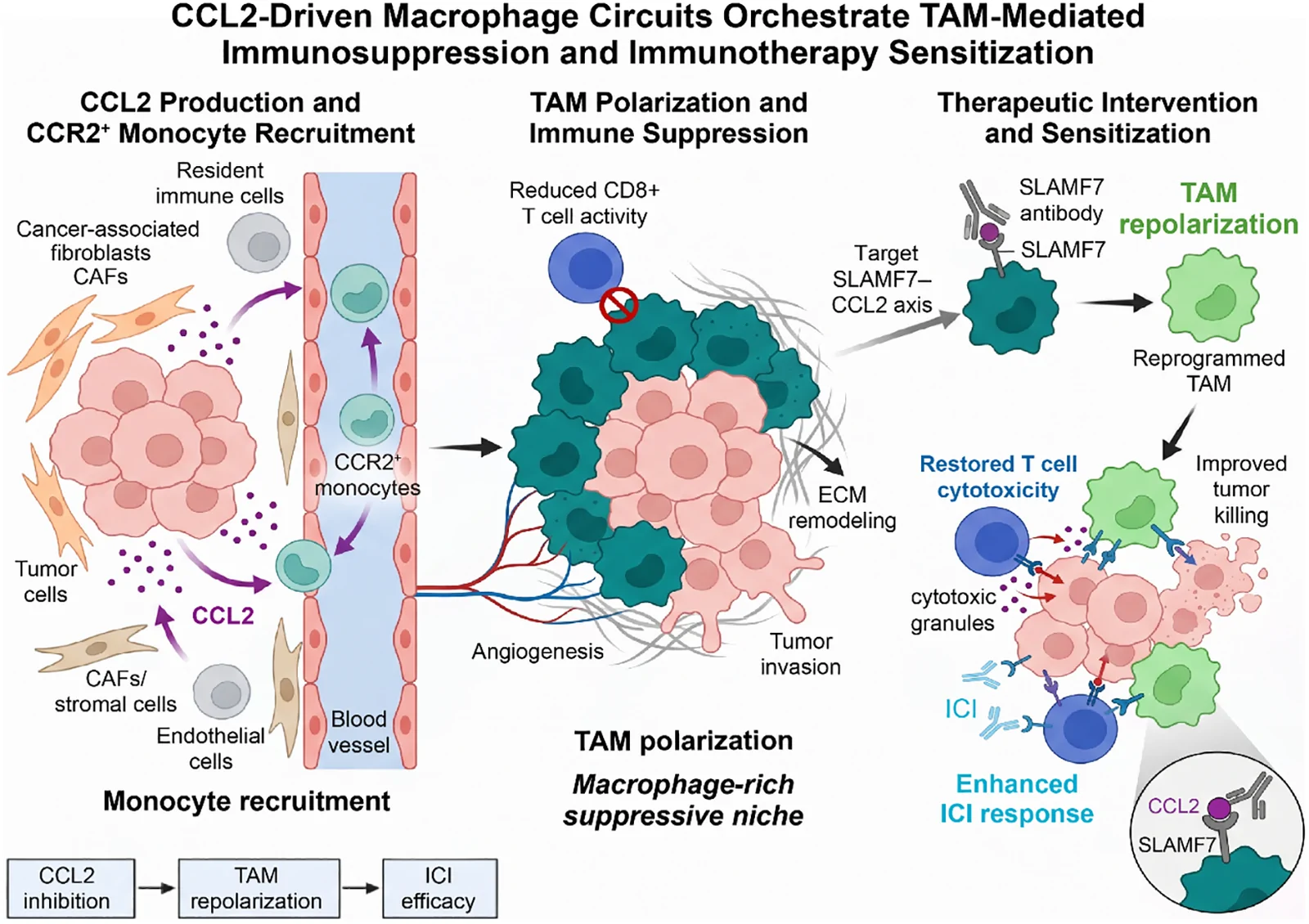
CCL2-associated macrophage circuits in immune suppression and immunotherapy sensitization. CCL2 produced by tumor, stromal, endothelial, fibroblast, and immune cells recruits CCR2^+^ monocytes into tumor tissues, where they differentiate into TAMs. These macrophages form suppressive niches that inhibit CD8^+^ T-cell activity and support angiogenesis, ECM remodeling, invasion, and therapy resistance. Targeting SLAMF7-regulated CCL2 signaling repolarizes TAMs and improves immunotherapy response.

## 8 Cross-axis comparison: convergent mechanisms of chemokine-mediated immune exclusion and therapy resistance

The chemokine–receptor axes discussed above differ in ligand source, receptor-expressing cells, tumor type, and therapeutic context. CXCL12–CXCR4 is strongly linked to stromal immune exclusion (29), CXCR2 ligand axes frequently organize neutrophil-rich suppressive niches (28), CCL20–CCR6 often supports Treg recruitment (30), CCL5–CCR5 can shape perivascular macrophage niches and adaptive resistance (31, 32), and CCL2-associated signaling regulates monocyte/macrophage recruitment and polarization (117). Despite these differences, several convergent mechanisms emerge. These axes repeatedly regulate four major features of tumor immunity: effector T-cell exclusion or dysfunction, suppressive myeloid/Treg accumulation, tumor-cell-intrinsic resistance programs, and spatial niche organization (1).

### 8.1 Chemokine axes converge on immune exclusion

Although individual chemokine axes recruit distinct immune-cell subsets, many converge on a common outcome: reduced productive contact between effector lymphocytes and malignant cells. This may occur through physical exclusion of T cells from tumor-cell nests, recruitment of suppressive cells that inhibit T-cell function, or induction of tumor-cell programs that reduce immune sensitivity (1). The CXCL12–CXCR4 axis represents the clearest example of spatial immune exclusion. In pancreatic cancer, CXCL12-rich stromal compartments can restrict effector T cells to fibroblast- or stroma-dominated regions, preventing their efficient entry into tumor-cell nests (29, 63). Clinical studies using CXCR4 antagonism in pancreatic cancer support the idea that disrupting this axis can increase T-cell recruitment and improve immune accessibility (127). However, these studies also show that increased T-cell entry does not automatically translate into durable antitumor immunity if compensatory suppressive myeloid programs emerge (55). The CXCL5–CXCR2 axis illustrates a different route to immune exclusion. Instead of creating a stromal barrier, tumor-derived CXCL5 can promote neutrophil-rich inflammatory niches that reduce effective T-cell infiltration or function. In obesity-associated pancreatic cancer, depletion of tumor-derived CXCL5 improves T-cell infiltration and enhances anti-PD-1 response, suggesting that CXCL5-driven chemokine programs can restrict productive lymphocyte entry or persistence in tumors (73). The CCL20–CCR6 axis also contributes to functional exclusion by recruiting CCR6^+^ Tregs. In pancreatic cancer, tumor-derived CCL20 attracts CCR6^+^ Tregs and reinforces an immunosuppressive microenvironment. Although this does not necessarily exclude T cells physically from the tumor, it can exclude effective T-cell function by creating a Treg-rich suppressive niche. Thus, immune exclusion should be understood broadly: it includes not only anatomical exclusion from tumor nests but also functional exclusion through suppressive cellular circuits. Taken together, these examples show that chemokine-mediated immune exclusion is not mediated by a single universal mechanism. Some axes create physical barriers, some recruit suppressive immune populations, and others reduce the functional quality of T-cell responses. The shared outcome is impaired effector lymphocyte access to malignant cells, either spatially or functionally.

### 8.2 Chemokine axes converge on suppressive myeloid/Treg accumulation

A recurring theme across chemokine systems is that tumors frequently use chemokine gradients to recruit or retain suppressive immune populations rather than cytotoxic lymphocytes. These suppressive populations include neutrophils, granulocytic myeloid-derived suppressor cells, tumor-associated macrophages, inflammatory monocytes, and regulatory T cells. Their accumulation can weaken antitumor immunity, support tumor growth, and reduce responsiveness to immune checkpoint blockade.

The CXCR2 ligand axes are strongly associated with neutrophil-dominated immune suppression. In NASH-associated HCC, CXCR2-dependent neutrophil recruitment links chronic metabolic inflammation to immune checkpoint resistance. In HCC more broadly, myeloid IL-8/CXCR2 signaling maintains suppressive myeloid programs that limit immunotherapy response (53). In lung cancer, CXCL5-mediated accumulation of mature neutrophils impairs anticancer CD8^+^ T-cell differentiation and limits the efficacy of checkpoint inhibitors. These studies support a model in which CXCR2 ligands convert inflammatory chemokine signals into neutrophil-rich suppressive niches.

The CCL20–CCR6 axis represents a Treg-centered suppressive module. Tumor-derived CCL20 in pancreatic cancer recruits CCR6^+^ Tregs, whereas galectin-1-induced TAMs in HCC secrete CCL20 to promote Treg recruitment (47, 87). These examples show that CCL20 can originate from either malignant cells or macrophages, but both routes converge on Treg accumulation and immune suppression. This flexibility allows different tumor ecosystems to activate the same suppressive lymphocyte recruitment program through distinct cellular sources. The CCL2-associated axis primarily shapes monocyte/macrophage accumulation and polarization. CCL2 recruits CCR2^+^ monocytes, which can differentiate into TAMs under tumor-derived cues (117). In HCC, SLAMF7-regulated CCL2 signaling maintains immunosuppressive macrophage states, and targeting this pathway repolarizes macrophages and sensitizes tumors to immunotherapy (54). This suggests that macrophage-associated chemokine circuits regulate not only myeloid-cell abundance but also TAM functional phenotype. The CCL5–CCR5 axis further illustrates how chemokines can organize suppressive myeloid compartments. In breast cancer, LYVE-1^+^ macrophages form a CCR5-dependent perivascular niche that influences CD8^+^ T-cell recruitment and chemotherapy response (31). Here suppressive immune remodeling is not simply a matter of total macrophage number; it depends on the spatial organization of macrophages within a vascular-associated niche.

Overall, these axes converge on a shared immunological principle: tumors often exploit chemokine systems to favor the recruitment, retention, or functional polarization of suppressive immune cells. This helps explain why inflammatory tumors are not always immunologically “hot” in a beneficial sense. A tumor may be highly inflamed but still resistant to therapy if the dominant inflammatory populations are neutrophils, TAMs, or Tregs rather than functional cytotoxic lymphocytes.

### 8.3 Chemokine axes regulate tumor-cell-intrinsic resistance programs

Chemokine–receptor signaling also acts directly on malignant cells. This tumor-cell-intrinsic dimension is essential because chemokines are often discussed only as immune-cell trafficking cues. However, many tumor cells express chemokine receptors and respond to chemokine stimulation by activating survival, invasion, immune-evasion, metabolic, or stress-adaptation pathways (128). The CXCL5–CXCR2 axis provides a clear example. In lung cancer, CXCL5 can upregulate PD-L1 expression through PXN/AKT signaling while also promoting neutrophil chemotaxis. This means that CXCL5 simultaneously modifies the immune microenvironment and directly increases a tumor-cell checkpoint molecule. Such dual activity allows a single chemokine axis to suppress antitumor immunity through both extrinsic and intrinsic mechanisms (50).

The CCL5–CCR5 axis also regulates tumor-cell survival under therapy pressure. In liver cancer, activation of the CCL5–CCR5–CYP1A1 pathway promotes survival during combined targeted and immune-based therapy. This finding suggests that chemokine signaling can engage stress-response or metabolic adaptation pathways that help malignant cells withstand treatment. In this setting, CCL5–CCR5 is not merely a recruitment axis; it becomes a tumor-cell survival circuit (32). Similarly, CXCR2 signaling can influence lung cancer cell behavior and chemotherapy response. Targeting CXCR2 suppresses lung cancer progression and enhances cisplatin efficacy, with effects on tumor-cell proliferation, apoptosis, senescence, EMT-related phenotypes, and tumor-associated inflammatory programs. These findings indicate that CXCR2-dependent signaling contributes to conventional treatment resistance as well as immunotherapy resistance (74). These examples support an important conceptual shift. Chemokines should not be viewed only as microenvironmental signals acting on immune cells. They can also function as direct regulators of malignant cell plasticity. Through tumor-cell-expressed receptors, chemokine axes may promote PD-L1 expression, EMT, survival signaling, metabolic adaptation, drug tolerance, and resistance to combined therapeutic pressure. Therefore, effective targeting of chemokine pathways may require attention to both immune-cell and tumor-cell receptor expression.

### 8.4 Chemokine axes define spatial niches

The most distinctive feature of chemokine biology is its spatial nature. Chemokines form gradients, and these gradients guide cell positioning within tissues. In tumors, this spatial function becomes highly consequential because immune-cell location determines whether immune responses remain peripheral, become suppressive, or directly engage malignant cells (1). The CCR5-dependent perivascular macrophage niche in breast cancer is a strong example of spatial chemokine organization. LYVE-1^+^ macrophages form a perivascular compartment regulated by CCR5-dependent signaling. This niche influences CD8^+^ T-cell recruitment and chemotherapy response. Importantly, the biological effect is not simply due to macrophage presence, but to their location around blood vessels, where they can regulate immune-cell entry, vascular function, and therapeutic access (31).

The CCL20–CCR6 axis in prostate cancer bone metastasis illustrates chemokine organization within a metastatic niche. Bone metastases are not passive sites of tumor growth; they are specialized tissue environments shaped by stromal, immune, and bone-resident cells. CCL20 enrichment in myeloid cells and CCR6 expression in T cells define a suppressive metastatic circuit. Disruption of this axis restores T-cell reactivity and improves survival in preclinical models, indicating that chemokine-defined metastatic niches can be therapeutically actionable (46). The CXCL12–CXCR4 axis in pancreatic cancer represents a stromal exclusion niche. CXCL12-rich stromal compartments help retain or redirect immune cells away from tumor-cell nests (29, 63). CXCR4 blockade can increase immune-cell access, but the immune outcome depends on the composition of recruited cells and the compensatory expansion of myeloid populations (55). This example demonstrates that spatial immune exclusion is not only a physical property of dense stroma but also a chemokine-guided positioning program.

Across these examples, chemokine gradients create functional compartments in which immune cells are either excluded from tumor nests or retained in suppressive stromal, perivascular, or metastatic niches. This spatial organization helps explain why bulk immune-cell abundance is often insufficient to predict therapy response. A tumor may contain T cells, macrophages, or neutrophils, but their impact depends on where they are located and which cells they contact (1). Therefore, future studies should move beyond measuring chemokine expression or immune-cell abundance alone. Spatial transcriptomics, multiplex imaging, imaging mass cytometry, spatial proteomics, and functional perturbation models are needed to determine how chemokine axes organize immune-cell neighborhoods and how these neighborhoods shape therapy response (129, 130).

### 8.5 Representative evidence from colorectal, gastric, and ovarian cancers

Although pancreatic cancer and hepatocellular carcinoma provide highly illustrative models for stromal immune exclusion and myeloid-dominated resistance, chemokine-driven immune remodeling is not restricted to these tumor types. In colorectal cancer, the CCL20–CCR6 axis has been linked to Treg recruitment, cancer stemness, metastasis, and chemoresistance—for example, colorectal cancer cell-derived CCL20 can recruit regulatory T cells and promote chemoresistance through FOXO1/CEBPB/NF-κB signaling, whereas RANKL/RANK signaling can recruit Tregs through the CCL20–CCR6 pathway and promote stemness and metastasis (131). The CCL5–CCR5 axis is also clinically relevant in colorectal cancer liver metastases, where CCR5 blockade with maraviroc has been associated with macrophage repolarization and antitumor immune remodeling in early clinical studies (132). Gastric cancer provides additional examples of chemokine-mediated immune suppression and checkpoint resistance. TAM-derived CXCL8 can promote immune evasion through PD-L1 expression in gastric cancer, and pharmacological CXCR1/2 inhibition has been reported to reduce PD-L1^+^ macrophages and promote antitumor immunity (133). The CCL20–CCR6 axis also participates in gastric tumor microenvironment remodeling, as PPARδ-driven CCL20 expression can recruit CCR6^+^ immune cells and promote gastric adenocarcinoma development (134, 135). More recently, CXCL12–CXCR4 signaling has been implicated in anti-PD-1 resistance in gastric cancer through PD-L1 upregulation and M2-like TAM polarization (136). Ovarian cancer further illustrates how chemokine circuits contribute to immune exclusion and treatment resistance. Cancer-associated mesenchymal stromal cells can drive CD8^+^ T-cell exclusion and anti-PD-L1 resistance through secretion of CCL2, CX3CL1, and TGF-β1, which recruit suppressive monocytic cells and promote immunosuppressive macrophage polarization (137). In addition, the MYBL2–CCL2 axis promotes ovarian cancer progression and anti-PD-1 resistance by inducing TAM recruitment and M2-like polarization, while CCL2 overexpression has been associated with paclitaxel resistance through both autocrine tumor-cell signaling and macrophage recruitment (138). These examples show that chemokine-driven immune exclusion and therapy resistance are shared principles across multiple tumor types, even though the dominant ligand–receptor axes and cellular sources differ by tissue context.

### 8.6 Integrated model: from chemokine gradients to resistant immune ecosystems

The cross-axis comparison reveals a common organizing principle: chemokine–receptor axes convert molecular gradients into cellular geography and functional immune states. Rather than simply controlling leukocyte entry into tumors, chemokine networks regulate immune-cell recruitment, retention, spatial positioning, cell–cell interactions, and functional differentiation within anatomically distinct tumor niches (1, 6). Spatially resolved studies further demonstrate that chemokine-associated immune-cell organization can generate distinct intratumoral cellular neighborhoods with different functional and clinical consequences (4).

Each axis may involve different ligands and responding cells, but many protumor chemokine circuits converge on resistant immune ecosystems characterized by poor effector T-cell access, accumulation of suppressive myeloid cells or Tregs, tumor-cell adaptive survival, and spatially organized immunosuppressive niches (1, 6). This integrated model can be summarized in four steps. First, tumor cells, stromal cells, endothelial cells, macrophages, or therapy-stressed tissues produce chemokines. Second, these chemokines recruit or retain specific receptor-expressing immune-cell subsets. Third, recruited cells are positioned within defined tumor regions such as stromal barriers, perivascular zones, metastatic niches, or neutrophil-rich suppressive areas. Fourth, these spatially organized populations can suppress effector immunity or support tumor-cell survival, thereby contributing to resistance to immune checkpoint blockade, chemotherapy, targeted therapy, or combination regimens (1, 6). This model also helps explain why single-axis blockade may produce incomplete therapeutic responses. In metastatic pancreatic cancer, CXCR4 antagonism increased intratumoral T-cell infiltration but was accompanied by enrichment of immunosuppressive macrophages, illustrating how disruption of one chemokine barrier can activate or reveal compensatory suppressive programs (55). Similarly, CXCR2 inhibition reduces neutrophil-dominated immune suppression and can sensitize inflammation-associated hepatocellular carcinoma to immune checkpoint blockade, supporting combinations that simultaneously relieve myeloid suppression and restore T-cell activity (53). Targeting CCL20–CCR6 can reduce CCR6^+^ Treg-associated immunosuppression, but other stromal and myeloid barriers may remain operative within the same tumor ecosystem (1, 8). CCL5–CCR5 signaling is likewise highly context-dependent: CCR5-dependent LYVE-1^+^ macrophages can form specialized perivascular niches that influence chemotherapy responses, whereas tumor-cell-intrinsic CCL5–CCR5 signaling can activate adaptive survival programs such as CYP1A1-associated resistance under therapeutic pressure (31, 32). Thus, the consequences of chemokine-axis blockade depend on the dominant receptor-expressing population, tumor genotype, spatial niche, treatment context, and compensatory chemokine circuits.

Accordingly, chemokine-targeted therapy should be developed as a circuit-reprogramming strategy rather than as a simple blockade of cell migration. The therapeutic objective should be to reorient tumor immune architecture by increasing productive effector T-cell access, reducing suppressive myeloid and Treg niches, weakening tumor-cell adaptive survival programs, and improving sensitivity to existing therapies (1). This cross-axis perspective provides a mechanistic foundation for the therapeutic strategies discussed in the next section. Spatially defined immune niches organized by chemokine–receptor axes are summarized in **Table 2**. These include stromal immune-exclusion compartments, neutrophil-rich suppressive regions, Treg-enriched niches, perivascular macrophage niches, metastatic immune niches, and macrophage-dominated resistance compartments.

**Table 2:** Spatial niches organized by chemokine-receptor axes in cancer.

Spatial niche	Chemokine-receptor axis	Key cellular components	Main spatial feature	Functional consequence	Niche-focused therapeutic note	Support level	3 PMID
Stromal immune-exclusion niche	CXCL12-CXCR4	Stromal cells, CAF-like compartments, CXCR4+ immune cells, T cells	T cells are retained in stromal regions with limited access to tumor-cell nests	Reduced immune-cell accessibility and poor checkpoint responsiveness	Spatial reversal of stromal exclusion by CXCR4 blockade has early clinical proof-of-concept; not an approved solid-tumor strategy	EC	32451495
CXCR4-remodeled pancreatic immune niche	CXCL12-CXCR4	T cells, macrophages, stromal compartments	CXCR4 antagonism increases immune-cell recruitment but can be accompanied by macrophage expansion	T-cell recruitment may be counterbalanced by suppressive myeloid accumulation	Suggests that spatial T-cell entry may need myeloid-aware combinations rather than CXCR4 blockade alone	EC/PE	40815609
Neutrophil-rich inflammatory HCC niche	CXCR2 ligand-CXCR2	Neutrophils, myeloid cells, T cells, HCC cells	CXCR2+ neutrophils accumulate in inflammation-associated HCC	Inflammatory but ICI-resistant immune architecture in NASH-HCC	CXCR2 inhibition experimentally reverses neutrophil-dominated checkpoint resistance in models	PE	35477863
Myeloid IL-8/CXCR2 suppressive niche	IL-8/CXCR2	Suppressive myeloid cells, HCC cells, T cells	Myeloid-enriched immune-suppressive compartment	Suppressed antitumor immunity and ICI resistance	IL-8/CXCR2 targeting has experimental support for weakening suppressive myeloid signaling	PE	39916327
Neutrophil-rich lung cancer niche	CXCL5-CXCR2	Lung cancer cells, mature neutrophils, CD8+ T cells	CXCL5-driven neutrophil accumulation within tumor tissues	Impaired CD8+ T-cell differentiation and reduced checkpoint inhibitor efficacy	CXCL5/CXCR2 targeting is experimentally supported as a way to reduce a neutrophil-rich suppressive niche	PE	35402081
PD-L1-associated CXCL5 immune-evasion niche	CXCL5-CXCR2 / PXN-AKT	Lung cancer cells, neutrophils, CD8+ T cells	CXCL5 links neutrophil chemotaxis with tumor-cell PD-L1 induction	CD8+ T-cell suppression through immune and tumor-cell-intrinsic mechanisms	PXN-AKT-PD-L1 regulation is reported in one mechanistic study; combination with PD-1/PD-L1 blockade remains exploratory	SS/HG	39034411
Obesity-shaped pancreatic immune niche	CXCL5-CXCR2	Pancreatic cancer cells, T cells, CXCR2+ myeloid cells	In one obese PDAC mouse model, host metabolic context was linked to CXCL5-dependent immune remodeling	Reduced T-cell infiltration and weaker anti-PD-1 response	CXCL5 depletion improved T-cell infiltration in a mouse model; human validation is needed before biomarker or patient-selection use	SS	40121029
Bone metastatic immune-suppressive niche	CCL20-CCR6	Myeloid cells, CCR6+ T cells, metastatic prostate cancer cells	Chemokine-mediated immune suppression within bone metastases	Reduced T-cell reactivity and metastatic immune escape	CCL20-CCR6 disruption experimentally restores T-cell reactivity in models	PE	34719426
Treg-rich pancreatic tumor niche	CCL20-CCR6	Pancreatic cancer cells, CCR6+ Tregs, effector T cells	Tumor-derived CCL20 recruits CCR6+ Tregs into the tumor microenvironment	Treg-mediated immune suppression	CCL20/CCR6 targeting is a preclinical strategy for reducing Treg-rich niches	PE	41643988
TAM-driven Treg niche in HCC	TAM-derived CCL20-CCR6	Galectin-1-induced TAMs, Tregs, HCC cells	TAM-derived CCL20 creates a Treg-enriched suppressive niche	Repression of antitumor immunity	Galectin-1 or CCL20-CCR6 targeting experimentally reduces Treg accumulation	PE	39853961
Perivascular macrophage niche	CCL5-CCR5	LYVE-1+ macrophages, blood vessels, CD8+ T cells, breast cancer cells	CCR5-dependent macrophages localize around blood vessels	Altered CD8+ T-cell recruitment and chemotherapy response	CCR5 blockade may disrupt a spatial macrophage niche; evidence is preclinical and niche-specific	PE	37442140
Genotype-associated immunosuppressive niche	CCL5-CCR5	BAP1-mutant ccRCC cells, CCR5+ immune cells	Tumor genotype shapes a CCR5-associated immune state	Immunosuppressive microenvironment	CCR5 blockade is a biomarker-linked hypothesis for selected genetic contexts and requires clinical validation	HG	32371459
Therapy-adaptive liver cancer niche	CCL5-CCR5-CYP1A1	Liver cancer cells, chemokine-producing microenvironmental cells	Chemokine signaling supports tumor-cell survival under treatment pressure	Resistance to targeted therapy plus immunotherapy	CCR5 blockade may weaken adaptive tumor-cell survival programs; CYP1A1 mechanism is preliminary single-study evidence	SS	39183447
Macrophage-dominated HCC niche	SLAMF7-regulated CCL2 signaling	Monocytes, TAMs, HCC cells, T cells	CCL2-associated circuit sustains suppressive macrophage states	TAM-mediated immunotherapy resistance	SLAMF7-CCL2 targeting experimentally repolarizes macrophages and sensitizes HCC to ICI	PE	38484085

## 9 Therapeutic targeting of chemokine–receptor axes

Therapeutic targeting of chemokine–receptor axes remains an emerging strategy rather than an established class of approved antitumor therapies (139). Although several chemokine-receptor antagonists have reached clinical use in non-solid-tumor, transplant-supportive, or infectious-disease contexts, their use as immune-modulatory cancer therapies remains investigational (59, 61, 140). Therapeutic targeting of chemokine–receptor axes offers an opportunity to remodel the tumor immune microenvironment rather than directly kill tumor cells alone. Because chemokine pathways regulate immune-cell trafficking, spatial localization, suppressive niche formation, and tumor-cell adaptation, their inhibition may convert immune-excluded or myeloid-rich tumors into more therapy-responsive states (2). However, the therapeutic value of chemokine targeting depends strongly on tumor context, dominant immune populations, spatial niche structure, and combination strategy. A key lesson from recent studies is that chemokine blockade should not be viewed simply as a way to stop immune-cell migration. It instead should be considered a strategy to reprogram tumor immune architecture (141). The therapeutic goal is to increase productive effector T-cell access, reduce suppressive myeloid and Treg accumulation, weaken tumor-cell adaptive resistance programs, and improve sensitivity to immune checkpoint blockade, chemotherapy, or targeted therapy (142). The therapeutic relevance of chemokine–receptor axes is summarized in **Table 3**, with emphasis on treatment context, resistance mechanisms, and potential intervention strategies. These examples support the concept that chemokine-targeted therapy should be developed as a combination strategy to overcome immune exclusion, suppressive-cell accumulation, and adaptive tumor-cell resistance (55).

**Table 3:** Chemokine–receptor axes associated with therapy resistance and therapeutic intervention.

Chemokine axis	Treatment context	Resistance mechanism	Therapeutic strategy	Key implication	Representative PMID
CXCL12–CXCR4	Preclinical PDAC checkpoint resistance	FAP+ CAF-derived CXCL12 excludes T cells from cancer-cell regions	CXCR4 inhibition (AMD3100) + anti-PD-L1	Foundational rationale for CXCR4 blockade combined with checkpoint inhibition	24277834
CXCL12–CXCR4	PD-1 blockade in metastatic pancreatic cancer	Stromal immune exclusion limits T-cell access to tumor-cell nests	CXCR4 antagonist + PD-1 blockade ± chemotherapy	Clinical translation of CXCR4-mediated immune-access strategy	32451495
CXCL12–CXCR4	PD-1 blockade in metastatic pancreatic cancer	T-cell recruitment may be accompanied by immunosuppressive macrophage accumulation	CXCR4 blockade + PD-1 blockade + myeloid-targeted therapy	CXCR4 inhibition may require macrophage-directed combinations	40815609
CXCR2 ligand–CXCR2	ICI resistance in NASH-HCC	CXCR2-dependent neutrophil recruitment creates an inflammatory but immunotherapy-resistant niche	CXCR2 inhibition + immune checkpoint blockade	Particularly relevant for inflammation-associated HCC	35477863
IL-8/CXCR2	Immunotherapy resistance in HCC	Myeloid IL-8/CXCR2 signaling sustains suppressive myeloid programs	IL-8/CXCR2 blockade + ICI	Targeting myeloid CXCR2 signaling may restore antitumor immunity	39916327
CXCL5–CXCR2	Checkpoint inhibitor resistance in lung cancer	CXCL5 recruits mature neutrophils that impair CD8+ T-cell differentiation	CXCL5/CXCR2 targeting + ICI	Neutrophil-rich tumors may benefit from CXCR2-axis inhibition	35402081
CXCL5–CXCR2	Immune escape in lung cancer	CXCL5 promotes neutrophil chemotaxis and upregulates PD-L1 via PXN/AKT signaling	CXCL5/CXCR2 blockade + PD-1/PD-L1 blockade	Targets both microenvironmental and tumor-cell-intrinsic immune resistance	39034411
CXCL5–CXCR2	Preclinical anti-PD-1 resistance in obesity-associated PDAC	Tumor-derived CXCL5 restricts T-cell infiltration in an obese mouse model	CXCL5 depletion + anti-PD-1; CXCR2-axis validation needed	Host metabolic status may shape chemokine-dependent ICI response, but evidence remains mainly preclinical and requires human validation	40121029
CXCR2-associated signaling	Cisplatin resistance in lung cancer	CXCR2 supports tumor progression, neutrophil infiltration, EMT, and survival programs	CXCR2 inhibition + cisplatin	CXCR2 blockade may sensitize tumors to chemotherapy	33814009
CCL20–CCR6	Chemoresistance in colorectal cancer	5-FU-resistant CRC cell-derived CCL20 recruits CCR6+ Tregs via FOXO1/CEBPB/NF-kB signaling	CCL20–CCR6 blockade + chemotherapy	May reduce Treg-driven chemoresistance	31395078
CCL20–CCR6	Stemness/metastasis-associated resistance in colorectal cancer	RANKL/RANK induces CCL20-mediated Treg recruitment and supports stemness and metastasis	Target RANKL/RANK–CCL20–CCR6 circuit + systemic therapy	Extends Treg-recruiting chemokine logic to CRC progression and resistance	38902257
CCL20–CCR6	Immune suppression in bone metastatic prostate cancer	Myeloid-derived CCL20 and CCR6+ T-cell interactions suppress T-cell reactivity	CCL20–CCR6 disruption	Metastatic chemokine niches may be therapeutically actionable	34719426
CCL20–CCR6	Immune suppression in pancreatic cancer	Tumor-derived CCL20 recruits CCR6+ Tregs	CCL20 blockade or CCR6 targeting	May reduce Treg-rich suppressive niches	41643988
TAM-derived CCL20–CCR6	Immune escape in HCC	Galectin-1-induced TAMs secrete CCL20 and recruit Tregs	Galectin-1 targeting; CCL20–CCR6 blockade	Upstream TAM regulators can be targeted to reduce Treg recruitment	39853961
CCL5–CCR5	Immune suppression in BAP1-mutant ccRCC	CCR5 signaling maintains an immunosuppressive immune state	CCR5 blockade ± ICI	Tumor genetic context may guide chemokine-targeted therapy	32371459
CCL5–CCR5	Colorectal cancer liver metastasis	CCR5-associated inflammatory cell exploitation and macrophage polarization at metastatic/invasive margins	Maraviroc-mediated CCR5 blockade	Provides early clinical oncology evidence for CCR5 blockade	27070705
CCL5–CCR5	Refractory MMRp/MSS metastatic colorectal cancer	Limited checkpoint responsiveness in immune-suppressive MSS CRC	Pembrolizumab + maraviroc	Feasible early clinical combination; efficacy likely requires biomarker selection	35427833
CCL5–CCR5	Chemotherapy response in breast cancer	CCR5-dependent perivascular macrophage niche influences CD8+ T-cell recruitment and treatment response	CCR5 blockade + chemotherapy	Spatial niche-targeted therapy may improve chemotherapy response	37442140
CCL5–CCR5–CYP1A1	Resistance to targeted therapy plus immunotherapy in liver cancer	CCL5/CCR5/CYP1A1 pathway supports tumor-cell survival under treatment pressure	CCR5 blockade + targeted therapy + immunotherapy	Chemokine signaling can directly support tumor-cell adaptive resistance	39183447
CXCL8–CXCR1/2	Immune evasion and checkpoint resistance in gastric cancer	TAM-derived CXCL8 promotes PD-L1 expression and PD-L1+ macrophage programs	CXCR1/2 inhibition (reparixin) ± ICI	May weaken TAM-associated immune evasion	30661053
CCL20–CCR6	Gastric adenocarcinoma immune remodeling	PPARd-driven CCL20 recruits CCR6+ immune cells and remodels the TME	PPARd inhibition or CCL20–CCR6 targeting	Representative gastric cancer chemokine-mediated immune suppression circuit	37572185
CXCL12–CXCR4	Anti-PD-1 resistance in gastric cancer	CXCL12/CXCR4/PI3K/AKT/HIF-1a signaling induces PD-L1 expression and M2-like TAM polarization	CXCL12–CXCR4 pathway targeting + anti-PD-1	Potential strategy to reverse gastric cancer ICI resistance	42310180
CCL2/CX3CL1/TGF-b1-associated circuit	Anti-PD-L1 resistance in ovarian cancer	CA-MSC-derived chemokines recruit suppressive monocytes and drive CD8+ T-cell exclusion	Hedgehog inhibition + anti-PD-L1; macrophage reprogramming	Demonstrates stromal chemokine-driven ICI resistance in ovarian cancer	34767446
MYBL2–CCL2	Anti-PD-1 resistance in ovarian cancer	MYBL2-high tumor cells induce CCL2-mediated TAM recruitment and M2-like polarization	Target MYBL2–CCL2 circuit + anti-PD-1	May enhance ICI efficacy by reducing immunosuppressive macrophages	37865750
CCL2-associated axis	Paclitaxel resistance in ovarian cancer	CCL2 overexpression promotes autocrine survival signaling and macrophage recruitment	CCL2/CCR2 targeting + paclitaxel	Links chemokine-driven macrophage recruitment to chemotherapy resistance	36076499
SLAMF7–CCL2 macrophage circuit	Immunotherapy resistance in HCC	CCL2-associated signaling maintains immunosuppressive macrophage states	SLAMF7-regulated CCL2 targeting + ICI	TAM repolarization may sensitize HCC to immunotherapy	38484085

### 9.1 CXCR4 blockade to overcome stromal immune exclusion

The CXCL12–CXCR4 axis is one of the most extensively investigated chemokine pathways for overcoming stromal immune exclusion. In pancreatic ductal adenocarcinoma (PDAC), CXCL12 produced by FAP^+^ cancer-associated fibroblasts can restrict effector T-cell access to malignant epithelial regions, and pharmacological CXCR4 inhibition can rapidly promote T-cell accumulation within tumor-cell areas and enhance the efficacy of PD-L1 blockade in preclinical models (29). Consistently, studies using human PDAC tissues and organotypic tumor slice cultures have shown that combined CXCR4 and PD-1 blockade promotes CD8^+^ T-cell mobilization toward juxtatumoral compartments and increases tumor-cell killing (63).

The COMBAT trial provided important clinical support for this therapeutic concept. In this phase IIa study of metastatic PDAC, the CXCR4 antagonist BL-8040 (motixafortide) was combined with pembrolizumab, with chemotherapy added in one cohort (58). CXCR4 blockade increased intratumoral CD8^+^ effector T-cell infiltration and reduced circulating or tumor-associated suppressive immune populations, while the combination cohort showed encouraging antitumor activity, including an objective response rate of 32% and disease control rate of 77% (58). These findings provided proof-of-concept evidence that targeting CXCR4 can remodel the immune microenvironment and potentially enhance the activity of chemotherapy and immune checkpoint blockade in otherwise poorly immunogenic PDAC. Nevertheless, these results should be interpreted as early-phase translational evidence rather than definitive clinical validation.

Subsequent clinical evidence further indicates that CXCR4 blockade may generate complex immune remodeling rather than a simple conversion from a “cold” to a “hot” tumor. In a phase II study combining the CXCR4 antagonist plerixafor with the PD-1 inhibitor cemiplimab in metastatic PDAC, treatment promoted intratumoral T-cell recruitment but was also associated with enrichment of immunosuppressive macrophage populations, while objective clinical activity remained limited (55). These findings suggest that disruption of CXCL12–CXCR4-mediated immune exclusion may uncover or induce compensatory myeloid suppressive programs. Therefore, improving T-cell accessibility alone may be insufficient unless macrophage-mediated immune suppression is simultaneously controlled. This observation provides a rationale for combining CXCR4 blockade with myeloid-directed therapies. CSF1/CSF1R inhibition can reprogram tumor-associated macrophages and improve responses to T-cell checkpoint blockade in pancreatic cancer models (143). Similarly, CCR2 blockade can reduce recruitment of inflammatory monocytes and tumor-associated macrophages; clinical evaluation of the CCR2 inhibitor PF-04136309 in combination with FOLFIRINOX has demonstrated the feasibility of this approach in patients with borderline resectable or locally advanced PDAC (121). Combined targeting of CCR2^+^ macrophages and CXCR2^+^ neutrophils further illustrates the potential benefit of simultaneously disrupting compensatory myeloid recruitment pathways (144). CD40 agonism represents another complementary strategy, capable of activating antigen-presenting cells, reprogramming macrophages, and remodeling pancreatic tumor stroma (145).

Thus, CXCR4 blockade should be viewed not merely as a strategy to increase immune-cell recruitment, but as one component of a broader immune-architecture reprogramming approach. Rational combinations may include CXCR4 antagonists with PD-1/PD-L1 blockade, chemotherapy, or myeloid-targeting interventions such as CSF1R inhibition, CCR2 blockade, or CD40 agonism. Importantly, future clinical studies should evaluate both effector T-cell redistribution and compensatory myeloid-cell dynamics, because increased lymphocyte access may not translate into durable therapeutic benefit if suppressive macrophage populations expand simultaneously (55).

### 9.2 CXCR2 inhibition to reduce neutrophil-mediated resistance

The CXCR2 ligand axis is a major therapeutic target in tumors dominated by neutrophils, granulocytic myeloid-derived suppressor cells, or IL-8/CXCL5-associated inflammation. CXCR2 ligands such as CXCL5 and CXCL8/IL-8 recruit suppressive myeloid populations that impair CD8^+^ T-cell function, promote immune checkpoint resistance, and support chemotherapy resistance. Therefore, CXCR2 inhibition may be particularly useful in tumors where neutrophil-rich inflammation is a major barrier to effective therapy. In NASH-associated HCC, CXCR2-dependent neutrophil recruitment links chronic metabolic inflammation to immune checkpoint resistance. CXCR2 inhibition can reprogram the neutrophil-rich inflammatory niche and restore immunotherapy responsiveness. This suggests that CXCR2 blockade may be especially relevant in inflammation-associated cancers, where chronic tissue injury creates a persistent myeloid-dominated immune landscape. In HCC more broadly, targeting myeloid IL-8/CXCR2 signaling has been shown to overcome immunotherapy resistance by reducing suppressive myeloid programs and restoring antitumor immune activity (69). This provides a translational rationale for combining CXCR2 inhibition with immune checkpoint blockade in HCC patients whose tumors show high IL-8/CXCR2 activity, neutrophil enrichment, or poor CD8^+^ T-cell infiltration. CXCR2 targeting may also enhance chemotherapy. In lung cancer, CXCR2 inhibition suppresses tumor progression and improves cisplatin efficacy. This effect may involve both reduction of tumor-associated neutrophil programs and direct modulation of tumor-cell phenotypes such as proliferation, apoptosis, senescence, and EMT. Thus, CXCR2 inhibition may serve as a sensitizing strategy not only for immunotherapy but also for conventional cytotoxic therapy. The CXCL5–CXCR2 axis is another important therapeutic entry point. In obesity-associated pancreatic cancer, depletion of tumor-derived CXCL5 improves T-cell infiltration and enhances anti-PD-1 response. In lung cancer, CXCL5 promotes neutrophil accumulation, impairs CD8^+^ T-cell function, and can increase PD-L1 expression through PXN/AKT signaling. These findings suggest that targeting CXCL5 or downstream CXCR2 signaling may improve checkpoint blockade efficacy in tumors where CXCL5-driven neutrophil recruitment or PD-L1 induction is prominent (28). Therapeutically, several strategies can be considered: CXCR2 inhibitors plus immune checkpoint inhibitors, CXCR2 inhibitors plus chemotherapy, CXCL5 or CXCL8 blockade plus anti-PD-1/PD-L1 therapy, and CXCR2-targeted approaches combined with T-cell-activating therapies. Biomarker selection will be essential. Tumors with high neutrophil infiltration, elevated CXCL5/CXCL8 expression, increased granulocytic MDSCs, or poor CD8^+^ T-cell activity may be most suitable for CXCR2-directed combinations.

### 9.3 Targeting CCL20–CCR6 to reduce Treg-dominated immune suppression

The CCL20–CCR6 axis represents a therapeutic opportunity in tumors characterized by Treg accumulation and suppressive lymphocyte niches. Unlike CXCR2 ligand axes, which are often neutrophil-centered, CCL20–CCR6 signaling frequently promotes recruitment of CCR6^+^ regulatory T cells or other suppressive lymphocyte populations (134). Therefore, targeting this axis may help reduce Treg-dominated immune suppression and improve effector T-cell activity (76). In prostate cancer bone metastasis, CCL20–CCR6 signaling contributes to an actionable immunosuppressive metastatic niche (146). Disruption of this axis restores T-cell reactivity and improves survival in preclinical models. This finding suggests that CCL20–CCR6 blockade may be particularly useful in metastatic sites where immune suppression is organized by local myeloid–T-cell communication (147). In pancreatic cancer, tumor-derived CCL20 recruits CCR6^+^ Tregs and exacerbates immunosuppression (47). This mechanism positions CCL20–CCR6 as a direct tumor-to-immune communication pathway (87). Targeting CCL20 production by malignant cells, blocking CCR6 on Tregs, or interfering with Treg recruitment may help reduce local immune tolerance (8). In HCC, galectin-1-induced TAMs secrete CCL20 and recruit Tregs. This finding expands the therapeutic logic beyond tumor-derived CCL20. It suggests that upstream macrophage regulators, such as galectin-1, may be targeted to reduce TAM-derived CCL20 production and suppress Treg recruitment. Therefore, therapeutic strategies may include direct CCL20 blockade, CCR6 targeting, inhibition of TAM-derived CCL20, or blockade of upstream regulators that induce CCL20 secretion (148). However, CCL20–CCR6 targeting requires caution. CCR6 is not exclusively expressed by Tregs; it can also be found on Th17 cells, memory T-cell subsets, B cells, and dendritic cell populations. Therefore, systemic CCR6 blockade may have complex immunological consequences (149). More precise strategies may require identifying tumors in which CCL20–CCR6 signaling specifically enriches suppressive Tregs rather than beneficial immune subsets. The most rational clinical application may be combination therapy. CCL20–CCR6 blockade could be paired with immune checkpoint inhibitors, Treg-depleting strategies, macrophage-reprogramming agents, or therapies that increase tumor antigen presentation (150). The goal would be to reduce Treg-mediated suppression while simultaneously enhancing effector T-cell priming and cytotoxic function.

### 9.4 CCR5 blockade and niche-targeted therapy

The CCL5–CCR5 axis is a context-dependent therapeutic target with relevance to immune remodeling, spatial niche organization, and tumor-cell adaptive resistance. Importantly, CCR5 blockade already has early clinical oncology experience through maraviroc, an orally available CCR5 antagonist approved for CCR5-tropic HIV-1 infection (132). In metastatic colorectal cancer, Halama and colleagues showed that CCR5 blockade with maraviroc could modulate the immune microenvironment of colorectal cancer liver metastases. Their study combined patient-derived functional models with a phase I trial in patients with advanced refractory colorectal cancer liver metastases and reported macrophage repolarization, mitigation of tumor-promoting inflammation, and objective tumor responses. More recently, the PICCASSO phase I trial evaluated pembrolizumab plus maraviroc in refractory mismatch repair-proficient/microsatellite-stable metastatic colorectal cancer, demonstrating that the combination was feasible and had a favorable toxicity profile, although clinical activity was limited to disease stabilization or responses in selected patients (151). These studies indicate that maraviroc provides a clinically tractable example of CCR5 blockade in oncology, but they also highlight the need for biomarker-guided patient selection and rational combination strategies.

Because CCR5 can be expressed by T cells, macrophages, Tregs, monocytes, and tumor cells, the effect of CCR5 blockade will depend on which cell population is dominant in a given tumor context. In BAP1-mutant clear cell renal cell carcinoma, CCR5 blockade enhances antitumor immunity (152). This finding suggests that tumor genetic context can define vulnerability to chemokine-receptor targeting. In such settings, CCR5 inhibition may convert an immunosuppressive tumor microenvironment into a more inflamed state. Therefore, patient selection based on molecular features such as BAP1 mutation may improve the therapeutic precision of CCR5-directed strategies. In breast cancer, CCR5-dependent signaling organizes LYVE-1^+^ macrophages into a perivascular niche that influences CD8^+^ T-cell recruitment and chemotherapy response. This example supports the concept of niche-targeted therapy (31). CCR5 blockade may not simply reduce macrophage migration; it may disrupt a spatially organized perivascular macrophage compartment that regulates immune-cell entry, vascular interactions, and treatment response. Therefore, spatial biomarkers, such as enrichment of LYVE-1^+^ macrophages around vessels or CCR5 activity within perivascular niches, may help identify patients who could benefit from CCR5-targeted therapy. In liver cancer, CCL5–CCR5 signaling can directly support tumor-cell survival under combined targeted and immune therapeutic pressure through the CCL5–CCR5–CYP1A1 pathway. This finding suggests that CCR5 blockade may also target tumor-cell-intrinsic adaptive resistance programs (32). In such cases, CCR5 inhibition could be combined with targeted therapy and immunotherapy to reduce tumor-cell survival under therapeutic stress. Therapeutic strategies involving CCR5 may include maraviroc-based CCR5 blockade in selected metastatic or immune-suppressed tumors, CCR5 blockade plus immune checkpoint inhibition, CCR5 blockade plus chemotherapy, or CCR5 inhibition combined with targeted and immune-based therapies (153, 154). However, because CCL5–CCR5 signaling can sometimes contribute to effector immune recruitment, indiscriminate blockade may not always be beneficial (155). Careful assessment of CCR5-expressing cell types, spatial niche structure, and tumor-cell dependence on CCR5 signaling will be essential.

### 9.5 CCL2-associated macrophage reprogramming

The CCL2/CCR2 axis is a classic pathway for recruiting inflammatory monocytes that can differentiate into tumor-associated macrophages (126). In many tumors, these macrophages acquire immunosuppressive phenotypes that limit T-cell activity, promote angiogenesis, support metastasis, and reduce immunotherapy efficacy. Therefore, CCL2-associated circuits are attractive targets in macrophage-rich tumors. However, therapeutic strategies aimed at this axis should not focus only on blocking monocyte recruitment (156, 157). TAMs are highly heterogeneous, and some macrophage populations may contribute to antigen presentation or antitumor inflammation under specific conditions. Therefore, macrophage reprogramming may be more effective than indiscriminate macrophage depletion. In HCC, SLAMF7-regulated CCL2 signaling maintains immunosuppressive macrophage states. Targeting this pathway repolarizes macrophages and sensitizes tumors to immunotherapy. This study provides a strong example of how chemokine-associated pathways can be therapeutically manipulated to alter macrophage function rather than simply reduce macrophage number. Potential strategies include CCL2/CCR2-targeted therapies, TAM repolarization approaches, SLAMF7-regulated CCL2 pathway inhibition, and combination with immune checkpoint blockade (158). The goal is to reduce macrophage-mediated suppression and create a microenvironment in which T cells can respond more effectively to immune checkpoint inhibitors. This approach may be especially relevant for tumors with high TAM infiltration, elevated CCL2 expression, poor T-cell function, and resistance to PD-1/PD-L1 blockade. Biomarkers such as macrophage abundance, CCL2 signaling activity, SLAMF7 expression, and spatial proximity between TAMs and T cells may help guide patient selection.

### 9.6 Principles for rational chemokine-targeted combinations

Across these therapeutic strategies, several principles emerge. First, chemokine-targeted therapy is most likely to be effective when matched to the dominant suppressive immune niche. CXCR4 blockade may be most suitable for stromal immune-excluded tumors, CXCR2 inhibition for neutrophil-rich tumors, CCL20–CCR6 targeting for Treg-dominated niches, CCR5 blockade for selected perivascular macrophage or genetically defined contexts, and CCL2-associated targeting for macrophage-rich tumors (49). Second, chemokine blockade will often require combination therapy. Because chemokine networks are redundant and compensatory, blocking one axis may lead to recruitment of alternative suppressive populations—for example, CXCR4 blockade can increase T-cell recruitment but may also be accompanied by suppressive macrophage accumulation. Therefore, chemokine inhibitors may need to be paired with immune checkpoint blockade, chemotherapy, targeted therapy, macrophage reprogramming, neutrophil inhibition, or Treg-targeted approaches. Third, spatial and cellular biomarkers are essential. Bulk chemokine expression alone may not predict therapeutic response. A high level of CCL5, CXCL5, or CCL2 does not reveal which cells produce the ligand, which cells express the receptor, where the interaction occurs, or whether the recruited immune cells are beneficial or suppressive. Spatial transcriptomics, multiplex immunostaining, single-cell profiling, and functional validation will be needed to guide therapeutic selection (159). Fourth, chemokine-targeted therapy should be understood as immune-architecture reprogramming. The aim is not simply to block migration but to reorganize tumor immune geography: increase effector T-cell access, reduce suppressive myeloid and Treg compartments, disrupt perivascular or metastatic immune niches, and weaken tumor-cell survival programs under treatment pressure. In summary, therapeutic targeting of chemokine–receptor axes has strong translational potential, but its success will depend on context-specific combination design and biomarker-guided patient selection. Future clinical development should move from single-axis inhibition toward rational remodeling of chemokine-controlled immune ecosystems (160).

### 9.7 Translational gap and clinical validation requirements

Although chemokine–receptor axes provide attractive therapeutic targets, an important translational gap remains. At present, no approved solid-tumor therapy has been established primarily on the basis of immune modulation through the chemokine–receptor mechanisms discussed in this review (141). Existing approved chemokine-receptor agents illustrate this gap: CXCR4 antagonists such as plerixafor and motixafortide are approved for hematopoietic stem-cell mobilization rather than antitumor immune remodeling (60, 161), whereas the CCR5 antagonist maraviroc is approved for CCR5-tropic HIV-1 infection rather than cancer therapy (162). Therefore, the current oncology evidence for CXCR4, CXCR2, CCR5, CCR6, and CCL2-related targeting should be interpreted mainly as preclinical, proof-of-concept, or early-phase translational evidence rather than as established clinical practice (163).

Several factors may explain why chemokine-receptor targeting has not yet translated into approved cancer therapies. First, chemokine networks are highly redundant, and blockade of one ligand–receptor pair may be bypassed by compensatory chemokines or parallel myeloid-recruitment pathways (164). Second, the same chemokine axis can exert opposite effects depending on whether the dominant receptor-expressing cells are suppressive myeloid cells, Tregs, tumor cells, effector T cells, or NK cells (2). Third, most trials have not prospectively selected patients using spatially resolved biomarkers of chemokine-driven immune exclusion or therapy resistance (1). Fourth, chemokine antagonists are unlikely to be sufficient as monotherapies in immune-cold or stromal-rich tumors and may require rational combinations with immune checkpoint blockade, chemotherapy, antiangiogenic therapy, radiotherapy, or macrophage-directed approaches (23). Finally, pharmacodynamic evidence is often incomplete; many studies show changes in circulating or bulk immune markers but do not prove that treatment disrupts a spatial chemokine gradient, restores effector-cell access to tumor nests, or reverses suppressive myeloid niches (55).

More decisive clinical validation would require biomarker-driven randomized trials that integrate clinical efficacy with mechanistic pharmacodynamic endpoints. These studies should prospectively define the target-enriched population, for example tumors with high stromal CXCL12, CXCR2-ligand-rich neutrophil niches, CCR5^+^ suppressive macrophage/Treg infiltration, or CCL2-associated TAM enrichment (1). They should demonstrate target engagement and immune remodeling through paired biopsies, spatial transcriptomics, multiplex immunofluorescence, imaging mass cytometry, flow cytometry, or circulating chemokine monitoring (165). Key pharmacodynamic readouts should include reduced suppressive myeloid-cell recruitment, increased CD8^+^ T-cell infiltration into tumor-cell regions, altered macrophage polarization, decreased Treg enrichment, and reversal of checkpoint-resistant spatial niches (166). Ultimately, these immune changes must be linked to clinically meaningful outcomes such as objective response rate, progression-free survival, overall survival, durability of response, and acceptable toxicity compared with standard regimens.

## 10 Challenges and future perspectives

Although chemokine–receptor axes provide an attractive framework for understanding immune–tumor communication, several conceptual and translational challenges remain. Chemokine systems are highly redundant, context-dependent, spatially regulated, and dynamically remodeled by therapy. Therefore, simple models that assign one chemokine axis to one immune-cell population or one tumor phenotype are unlikely to capture the full complexity of tumor immune ecology. Future studies should move beyond descriptive ligand–receptor mapping and toward spatially resolved, functionally validated, and therapeutically actionable chemokine circuit models.

### 10.1 Chemokine axes are context-dependent

One of the major challenges in the field is the context dependency of chemokine–receptor signaling. The same chemokine axis may have different, or even opposing, effects depending on tumor type, disease stage, tissue site, inflammatory state, treatment context, and receptor-expressing cell population. This complexity is particularly important when considering chemokine-targeted therapy.

The CCL5–CCR5 axis is a clear example. In some inflammatory contexts, CCL5–CCR5 signaling may contribute to effector T-cell recruitment and immune activation (167). However, in many tumors, it may promote tumor-associated macrophage accumulation, regulatory T-cell recruitment, metastatic progression, perivascular niche formation, or adaptive resistance (168, 169). Therefore, CCR5 blockade may be beneficial in selected immune-suppressive or genetically defined tumors, but it may not be universally advantageous across all cancer types (170). Similarly, the CCL2/CCR2 axis is often associated with monocyte recruitment and TAM accumulation, but the recruited monocyte compartment is heterogeneous (171). Some CCR2^+^ monocytes may differentiate into immunosuppressive macrophages, whereas others may acquire inflammatory or antigen-presenting functions under different microenvironmental conditions (124). As a result, simply blocking CCL2/CCR2 may not always produce predictable immune benefits. The therapeutic effect will depend on which monocyte/macrophage subsets are dominant and how they are programmed after entering the tumor.

The CXCL12–CXCR4 axis also illustrates this complexity (172). CXCR4 blockade can improve T-cell recruitment and reduce stromal immune exclusion, especially in pancreatic cancer (62). However, clinical studies suggest that CXCR4 antagonism may also be accompanied by compensatory accumulation of immunosuppressive macrophages. This means that increasing T-cell access is not sufficient if other suppressive immune compartments expand at the same time. These examples highlight a central principle: chemokine axes should not be interpreted in isolation. Their biological consequences depend on the broader immune ecosystem in which they operate. Future studies should therefore define not only which chemokines are expressed, but also which cells produce them, which cells respond to them, where the interaction occurs, and what functional state is induced in the responding cells (7).

### 10.2 Ligand–receptor inference requires benchmarking-aware interpretation and functional validation

Single-cell RNA sequencing and computational ligand–receptor inference have greatly expanded our ability to map potential communication networks in tumors. Commonly used tools and frameworks, such as CellPhoneDB, CellChat, NicheNet, LIANA, and related methods, can identify putative interactions between chemokine-producing and receptor-expressing cells, generate hypotheses about immune–tumor crosstalk, and reveal candidate axes associated with immune exclusion or therapy resistance (173–176). However, published benchmarking and comparative evaluation studies show that inferred communication networks can vary substantially depending on the ligand–receptor database, scoring strategy, expression threshold, statistical model, and whether downstream target-gene activity or spatial proximity is incorporated (177, 178). Therefore, ligand–receptor co-expression should not be interpreted as definitive proof of functional communication (179). There are several reasons for this limitation. First, mRNA expression does not always correlate with protein abundance. A chemokine transcript detected in one cell population may not result in sufficient secreted protein to form a functional gradient. Similarly, receptor mRNA expression does not guarantee receptor protein expression, surface localization, or signaling competence (180). Second, chemokines act in a spatially constrained manner. Even if a ligand and a receptor are expressed in the same tumor sample, the corresponding cells may not be physically close enough for meaningful communication (181, 182). Third, receptor engagement does not necessarily produce a functional biological effect unless downstream signaling is activated and alters cell behavior.

Several benchmarking studies provide practical guidance for interpreting these tools. Dimitrov et al. compared methods and ligand–receptor resources for cell–cell communication inference and developed LIANA as a framework to separate method-specific scoring from prior interaction resources, highlighting that both algorithm choice and database choice can influence the inferred communication landscape (176). Wang et al. systematically evaluated ligand–receptor-based cell–cell interaction inference tools and emphasized differences in sensitivity, specificity, robustness, and dependence on reference ligand–receptor databases (177). Xie et al. further benchmarked commonly used cell–cell interaction prediction tools using scRNA-seq data and showed that different tools may be preferable depending on whether the user prioritizes sensitivity, specificity, speed, or consensus-supported interactions (183). More recently, the ESICCC framework evaluated 18 ligand–receptor inference methods and five ligand/receptor-target inference methods across 116 datasets, including spatial transcriptomics, perturbation, proteomic, and scRNA-seq datasets, and provided decision-tree-style guidance for tool selection. These studies support a practical recommendation: candidate chemokine–receptor interactions should ideally be prioritized only when they are reproducible across complementary methods, supported by appropriate ligand–receptor resources, and consistent with spatial, protein-level, or perturbation-based evidence (178).

Therefore, ligand–receptor inference should be regarded as a hypothesis-generating and prioritization strategy, not as definitive proof of immune communication. For practical analysis, authors should report the tool and version used, the ligand–receptor database, filtering thresholds, cell-type annotation strategy, and whether results were confirmed by alternative methods or spatial information. Robust validation then requires multiple complementary approaches. Spatial proximity should be assessed using spatial transcriptomics, multiplex immunofluorescence, imaging mass cytometry, CODEX, or related methods. Protein expression should be validated by immunostaining, flow cytometry, mass cytometry, ELISA, proteomics, or spatial proteomic approaches. Functional relevance should be tested through ligand or receptor blockade, genetic perturbation, ex vivo organoid–immune coculture systems, animal models, or clinically annotated patient samples. This point is especially important for translational research. A study based only on public transcriptomic data may identify a putative chemokine axis, but without independent validation, it remains unclear whether the axis is functional, spatially relevant, or therapeutically targetable. Future work should combine computational inference with experimental perturbation and clinical validation to distinguish true communication circuits from correlative expression patterns.

### 10.3 Spatial validation is essential

Chemokine biology is inherently spatial. Chemokines act through gradients, local retention, receptor engagement, and tissue compartmentalization. Therefore, bulk measurements of chemokine expression are often insufficient to explain their function (129). A tumor with high CXCL12, CXCL5, CCL20, CCL5, or CCL2 expression may show very different immune phenotypes depending on where the chemokine is produced and which cells are positioned nearby. Bulk RNA sequencing cannot determine whether CD8^+^ T cells are located inside tumor-cell nests or trapped in stromal regions (184). It cannot reveal whether macrophages are diffusely distributed or organized around blood vessels. It cannot identify whether Tregs are clustered near tumor cells, antigen-presenting cells, or stromal barriers. These spatial differences are not minor details; they can determine whether an immune response is effective or suppressed.

Spatial validation is therefore essential for understanding chemokine-mediated immune exclusion and niche formation. Spatial transcriptomics can map chemokine and receptor expression across tissue regions (185). Multiplex immunofluorescence can define immune-cell localization relative to tumor cells, vessels, stroma, and extracellular matrix. CODEX and imaging mass cytometry can profile many immune and stromal markers simultaneously while preserving tissue architecture (186). Spatial proteomics can further clarify whether inferred ligand–receptor interactions are supported at the protein level. These approaches can together distinguish between inflamed tumors with productive T-cell infiltration and inflamed tumors dominated by suppressive myeloid or Treg niches.

Spatial validation is also important for therapy development—for example, CXCR4 blockade may be most useful in tumors where T cells are excluded from tumor nests by CXCL12-rich stromal compartments. CXCR2 inhibition may be most useful in tumors where neutrophils are spatially positioned to suppress T-cell activity. CCR5 blockade may be most relevant when macrophages form perivascular niches that control immune-cell access or chemotherapy response (187). Without spatial information, these therapeutic opportunities may be missed or misclassified.

Future studies should therefore integrate spatial data into biomarker development. Instead of measuring only total chemokine expression, investigators should assess ligand source, receptor-expressing cell type, immune-cell localization, spatial proximity, and treatment-induced changes in tissue architecture.

### 10.4 Therapeutic timing and combination are critical

Another major challenge is determining how and when to target chemokine axes therapeutically. Chemokine networks are dynamic and can change during tumor progression, metastatic colonization, and treatment. A chemokine axis that promotes immune suppression at baseline may be altered by chemotherapy, radiotherapy, targeted therapy, or immune checkpoint blockade (188). Conversely, therapy-induced inflammation may create new chemokine gradients that recruit either effector immune cells or suppressive myeloid populations.

Therapeutic timing is therefore critical. Blocking a chemokine axis too early or too broadly may interfere with beneficial immune recruitment, whereas blocking it at the appropriate stage may prevent suppressive niche formation—for example, CXCR4 blockade may increase T-cell recruitment in stromal immune-excluded tumors, but it may need to be combined with macrophage-directed therapy if suppressive macrophages accumulate after treatment. CXCR2 inhibition may be most effective in tumors with high neutrophil infiltration or CXCL5/CXCL8 activity, particularly when paired with immune checkpoint blockade or chemotherapy. CCR5 targeting may require patient selection based on genetic background, such as BAP1 mutation, or spatial features such as CCR5-dependent perivascular macrophage niches (189).

Combination therapy is likely to be essential because chemokine networks are redundant. Blocking one axis may allow another suppressive pathway to compensate (139, 190)—for example, reducing neutrophil recruitment through CXCR2 inhibition may not fully overcome Treg accumulation driven by CCL20–CCR6 or macrophage suppression driven by CCL2-associated circuits (30, 53, 54). Similarly, increasing T-cell infiltration through CXCR4 blockade may not be sufficient if T cells remain dysfunctional or if tumor cells activate checkpoint or survival pathways (55). Rational combinations may include chemokine-axis blockade with immune checkpoint inhibitors, chemotherapy, targeted therapy, Treg-directed approaches, macrophage reprogramming, neutrophil inhibition, stromal modulation, or cancer vaccines (23). The optimal combination should be selected according to the dominant resistance mechanism in each tumor. This requires biomarkers that capture not only chemokine expression, but also immune-cell composition, spatial niche structure, receptor-expressing populations, and functional immune states (1, 6). Thus, future therapeutic development should move from a one-size-fits-all model to context-guided chemokine intervention (142). The key question is not simply whether a chemokine axis is present, but whether it is the dominant driver of immune exclusion or therapy resistance in a given patient.

### 10.5 Future direction: from single-axis targeting to chemokine circuit reprogramming

The future of chemokine-targeted therapy may not lie in blocking a single chemokine pair but in reprogramming chemokine circuits that coordinate spatial immune architecture and therapy adaptation. Tumors rarely depend on one isolated ligand–receptor interaction. They instead activate interconnected chemokine networks involving tumor cells, stromal cells, endothelial cells, macrophages, neutrophils, Tregs, and effector lymphocytes. These networks evolve during tumor progression and under therapeutic pressure (1). A circuit-based view would integrate several dimensions. First, it would identify the dominant chemokine sources, such as tumor cells, CAFs, TAMs, endothelial cells, or therapy-injured tissue. Second, it would define the receptor-expressing target populations, including CD8^+^ T cells, Tregs, neutrophils, monocytes, macrophages, or tumor cells. Third, it would map spatial organization, such as stromal exclusion zones, perivascular macrophage niches, neutrophil-rich suppressive regions, or metastatic immune niches (31, 46). Fourth, it would determine functional consequences, including T-cell exclusion, myeloid suppression, Treg accumulation, PD-L1 induction, tumor-cell survival, or treatment adaptation. This approach would shift chemokine targeting from simple inhibition to immune-architecture engineering. The therapeutic aim would be to dismantle suppressive chemokine circuits while preserving or enhancing chemokine programs that support effector immune-cell recruitment—for example, an ideal strategy might simultaneously reduce CXCR2-driven neutrophil recruitment, prevent CCL20-mediated Treg accumulation, and enhance T-cell entry into tumor nests (28, 30). In other settings, therapy might combine CXCR4 blockade to open stromal barriers with macrophage reprogramming to prevent compensatory suppression (55). Future research should also prioritize longitudinal sampling. Tumor chemokine networks before therapy may differ from those after treatment initiation or at acquired resistance. Serial biopsies, circulating biomarkers, spatial profiling, and single-cell analysis of pre-treatment and post-treatment samples will be important for understanding how chemokine circuits evolve (191, 192). This is especially relevant for immunotherapy, where early immune remodeling may determine long-term response or resistance (193). Finally, artificial intelligence and network modeling may help integrate complex chemokine data across single-cell, spatial, proteomic, imaging, and clinical datasets. Such approaches could identify dominant communication circuits, predict compensatory pathways, and guide rational combinations (194, 195). However, computational predictions must be validated experimentally and clinically (178). In summary, chemokine–receptor axes provide a powerful framework for understanding immune–tumor communication, but their translational potential will depend on context-aware, spatially validated, and functionally tested models. The next phase of the field should move beyond cataloguing ligand–receptor pairs toward designing strategies that reprogram chemokine-controlled immune ecosystems and overcome therapy resistance.

## 11 Conclusions

Chemokine–receptor axes are central regulators of immune–tumor communication. Rather than functioning only as leukocyte trafficking signals, these axes organize the spatial and functional architecture of the tumor microenvironment. They determine which immune cells are recruited, where these cells are positioned, and whether they support antitumor immunity or reinforce immune escape. Through this multilayered regulation, chemokine signaling contributes to stromal immune exclusion, neutrophil- and macrophage-dominated suppressive niches, Treg accumulation, metastatic immune remodeling, and therapy-resistant tumor-cell states. The evidence discussed in this review highlights several representative chemokine modules. The CXCL12–CXCR4 axis serves as a prototype of stromal immune exclusion, particularly in pancreatic cancer. CXCR2 ligand axes, including CXCL5–CXCR2 and IL-8/CXCR2 signaling, drive neutrophil-rich suppressive niches and resistance to immune checkpoint blockade and chemotherapy. The CCL20–CCR6 axis promotes Treg recruitment and metastatic immune suppression. The CCL5–CCR5 axis remodels immune states, organizes perivascular macrophage niches, and supports adaptive resistance. CCL2-associated macrophage circuits regulate monocyte recruitment, TAM polarization, and immunotherapy responsiveness. Therapeutically, targeting chemokine–receptor axes offers a strategy to reprogram tumor immune architecture. However, successful translation will require more than blocking individual ligand–receptor pairs. Because chemokine functions are context-dependent and spatially organized, future approaches should integrate single-cell profiling, spatial validation, functional perturbation, and biomarker-guided patient selection. Rational combinations with immune checkpoint blockade, chemotherapy, targeted therapy, macrophage reprogramming, or neutrophil/Treg-directed strategies will likely be necessary. Overall, chemokine–receptor axes should be viewed as spatial communication circuits that coordinate immune-cell trafficking, tissue localization, suppressive niche formation, and tumor-cell adaptation. Understanding and therapeutically reprogramming these circuits may provide new opportunities to overcome immune exclusion and treatment resistance in cancer.
